# “*Reframing*” dopamine signaling at the intersection of glial networks in the aged Parkinsonian brain as innate *Nrf2/Wnt* driver: Therapeutical implications

**DOI:** 10.1111/acel.13575

**Published:** 2022-03-09

**Authors:** Bianca Marchetti, Carmela Giachino, Cataldo Tirolo, Maria F. Serapide

**Affiliations:** ^1^ Department of Biomedical and Biotechnological Sciences (BIOMETEC) Pharmacology Section Medical School University of Catania Catania Italy; ^2^ OASI Research Institute‐IRCCS, Troina (EN), Italy Troina Italy

**Keywords:** dopamine signaling, glial–neuron crosstalk, inflammation, Nrf2/Wnt signaling, oxidative stress, Parkinson's disease

## Abstract

Dopamine (DA) signaling via G protein‐coupled receptors is a multifunctional neurotransmitter and neuroendocrine–immune modulator. The DA nigrostriatal pathway, which controls the motor coordination, progressively degenerates in Parkinson's disease (PD), a most common neurodegenerative disorder (ND) characterized by a selective, age‐dependent loss of substantia nigra pars compacta (SNpc) neurons, where DA itself is a primary source of oxidative stress and mitochondrial impairment, intersecting astrocyte and microglial inflammatory networks. Importantly, glia acts as a preferential neuroendocrine–immune DA target, in turn, counter‐modulating inflammatory processes. With a major focus on DA intersection within the astrocyte–microglial inflammatory network in PD vulnerability, we herein first summarize the characteristics of DA signaling systems, the propensity of DA neurons to oxidative stress, and glial inflammatory triggers dictating the vulnerability to PD. Reciprocally, DA modulation of astrocytes and microglial reactivity, coupled to the synergic impact of gene–environment interactions, then constitute a further level of control regulating midbrain DA neuron (mDAn) survival/death. Not surprisingly, within this circuitry, DA converges to modulate *nuclear factor erythroid 2*‐*like 2* (Nrf2), the master regulator of cellular defense against oxidative stress and inflammation, and *Wingless* (*Wnt*)/*β*‐*catenin* signaling, a key pathway for mDAn neurogenesis, neuroprotection, and immunomodulation, adding to the already complex “signaling puzzle,” a novel actor in mDAn–glial regulatory machinery. Here, we propose an autoregulatory feedback system allowing DA to act as an endogenous *Nrf2*/*Wnt* innate modulator and trace the importance of DA receptor agonists applied to the clinic as immune modifiers.

## Graphical abstract

Besides its central role as neurotransmitter and neuroendocrine–immune modulator, we herein propose DA acting as endogenous Nrf2/Wnt innate modulator intersecting astrocyte–microglial inflammatory networks in PD vulnerability. With age and PD, the dramatic loss of DA‐mediated signaling at central and peripheral levels drives interacting harmful cascades whereby loss of DA/Nrf2/Wnt‐resilient signaling engenders a detrimental vicious cycle, further aggravating inflammation with severe outcome.

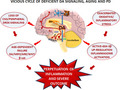

**Abbreviations**
ARE: antioxidant response elements
APC: adenomatous polyposis coli
α‐syn: α‐synuclein
βArr2: β‐Arrestin 2
cAMP: cyclic adenosine monophosphate
CK1α: casein kinase 1α
COMT: catechol‐O‐methyl transferase (COMT),
CPu: corpus striatum
CRD: cysteine‐rich domain
CREB: cAMP‐response element binding protein
CRYAB: α‐beta‐cristallin
CSF: cerebrospinal fluid
DA: dopamine
DAn: dopaminergic neurons
DAT: dopamine transporter
DOPAL: 3,4‐dihydroxyphenylacetaldehyde
DOPAC: 3,4‐dihydroxyphenylacetic acid
DRD1‐DRD5: dopamine receptors D1‐D5
Dvl: Dishevelled
Fzd: Frizzled
Fizz1: found in inflammatory zone 1
GFAP: glial fibrillary acidic protein
GDNF: glial‐derived neurotrophic factor
G6PD: glucose‐6‐phosphate dehydrogenase
GPCRs: G‐protein coupled receptors
GSH: Glutathione
GSK‐3β: glycogen synthase kinase 3β
Hmox1: heme oxygenase 1
HPG: hypothalamic‐hypophysealgonadal
HPA: hypothalamic‐hypophyseal‐adrenocortical axis
HVA: homovanilic acid
IBA1: ionized calcium‐binding adapter molecule 1
iNOS: inducible nitric oxide synthase
IL‐1 β: interleukin‐1β
IL‐6: interleukin‐6
Keap1: Kelch‐like ECH‐associated protein 1
L‐DOPA: levodopa (L‐3,4‐dihydroxyphenylalanine)
LEF: lymphoid enhancer binding factor
LHRH: luteinizing‐hormone releasing hormone
LPS: lipopolysaccharide
LRP: low‐density lipoprotein receptor‐related protein
LRRK2: leucine‐rich repeat kinase 2
ME: median eminence
mDAn: midbrain dopaminergic neurons
MAO‐A/B: monoamino oxidase‐A/B
MPP^+^
: 1‐methyl‐4‐phenylpyridinium
MPTP: 1‐methyl‐4‐phenyl‐1,2,3,6‐tetrahydropyridine
mtDNA: mitochondrial DNA
ND: neurodegenerative disorder
NF‐ĸB: nuclear factor kappa‐light‐chain‐enhancer of activated B cells
NLRP3: Nod‐like receptor protein
nNOS: neuronal nitric oxide synthase
Nrf2: nuclear factor erythroid‐2‐related factor 2
NSC: neural stem cell
Nurr1: nuclear receptor‐related factor 1
PD: Parkinson’s disease
PPP: pentose phosphate pathway
PHOX: phagocyte oxidase
PI3K: phosphoinositide 3‐kinase
PINK1: PTEN‐induced putative kinase
PNS: peripheral nervous system
PP2A: protein phosphatase‐2A
PRL: prolactin
RNS: reactive nitrogen species
ROS: reactive oxygen species
SN: substantia nigra
SNpc: substantia nigra pars compacta
6‐OHDA: six hdroxy‐dopamine
SVZ: subventricular zone
TAB1: transforming growth factor beta 1
TH: tyrosine hydroxylase
TIDA: tubero‐infundibular dopaminergic
3‐MT: 3‐methoxytyramine
3‐NT: 3‐nitrotyrosine
TNF‐α: tumor necrosis factor α
VMAT: vesicular monoamine transporter
VM: ventral midbrain
VTA: ventral tegmental area
Wnt1: wingless‐type mouse mammary tumor virus integration site1

## 1 INTRODUCTION

Dopamine (DA) is a central player in movement regulation, reward, and neuroendocrine–immune homeostasis. In the nigrostriatal pathway, the substantia nigra (SN, A9) cell bodies are responsible for the production and release of DA into the corpus striatum (Str), which governs motor coordination. In Parkinson's disease (PD), a most prevalent age‐dependent movement disorder and the second most common neurodegenerative disease (ND) affecting 2%–3% of the population >65 years of age, a selective and progressive loss of SN pars compacta (SNpc) neurons, associated with a slow degeneration of their terminals in the Str, gradually impairs motor function leading to the classical motor features of PD (i.e., bradykinesia, rest tremor, rigidity, and postural instability) (Obeso et al., 2017). A major pathological feature of PD is the presence of aggregates that localize in neuronal cytoplasm as Lewy bodies, mainly composed of α‐synuclein (α‐syn) and ubiquitin (Chu et al., 2019; Killinger & Kordower, 2019; Litvan et al., 2007; Ulusoy & Monte, 2013).

Remarkably, Parkinson's disease is the fastest growing neurological disorder in the world, with the number of patients affected expected to grow exponentially from almost 7 million in 2015 to >14.2 million in 2040. Such a Parkinson “pandemic” facing, now, the coronavirus disease 2019 (COVID‐19) pandemic is expected to cause a most severe health care, and social and economic burden (Dorsey et al., 2018; Helmich & Bloem, 2020). Particularly, COVID‐19 infection (Huang et al., 2020) intersects the pivotal environmental hallmarks for PD and other NDs, namely aging (Gerashchenko et al., 2020), chronic stress, and exacerbated inflammatory response (the so‐called “cytokine storm”) (Delgado‐Roche & Mesta, 2020; Huang et al., 2020), representing conditions recognized to drive and/or worsen Parkinson's symptoms.

Indeed, aging, a most dangerous vulnerability factor for PD, by promoting a sustained inflammatory activation of the glial cell compartment, for example, astrocytes and microglia, acts as critical “*vicious*” mechanism contributing to the onset and/or progression of the disease (Betarbet et al., 2002; Di Monte et al., 2002; Gao & Hong, 2011; Gao et al., 2011; Hirsch & Hunot, 2009; Marchetti & Abbracchio, 2005; McGeer & McGeer, 2008; Przedborski, 2010; Tu et al., 2019; Tu et al., 2019; Whitton, 2010; Zhu et al., 2021).

Regrettably, the underlying causes linking these pathological hallmarks with neurodegeneration still remain unclear, and by the time clinical manifestations appear, about 70% of the dopamine (DA) fibers in the caudate putamen (CPu) and almost 50% of the midbrain dopaminergic neurons (mDAns) in SNpc are already lost (Litvan et al., 2007; Obeso et al., 2017). The progression of the disease is slow in most cases, but irreversible, with current therapies (e.g., L‐3,4‐dihydroxyphenylalanine, L‐DOPA, the mainstay in PD treatment), being directed toward the replacement of DA levels in the brain, and, as such, provided only symptomatic relief (Jankovic, 2019; Schapira et al., 2014). Of note, these drugs do not modify the progressive neurodegenerative cell loss associated with PD that, in many cases, results in debilitating side effects (see Obeso et al., 2017).

**FIGURE 1:**
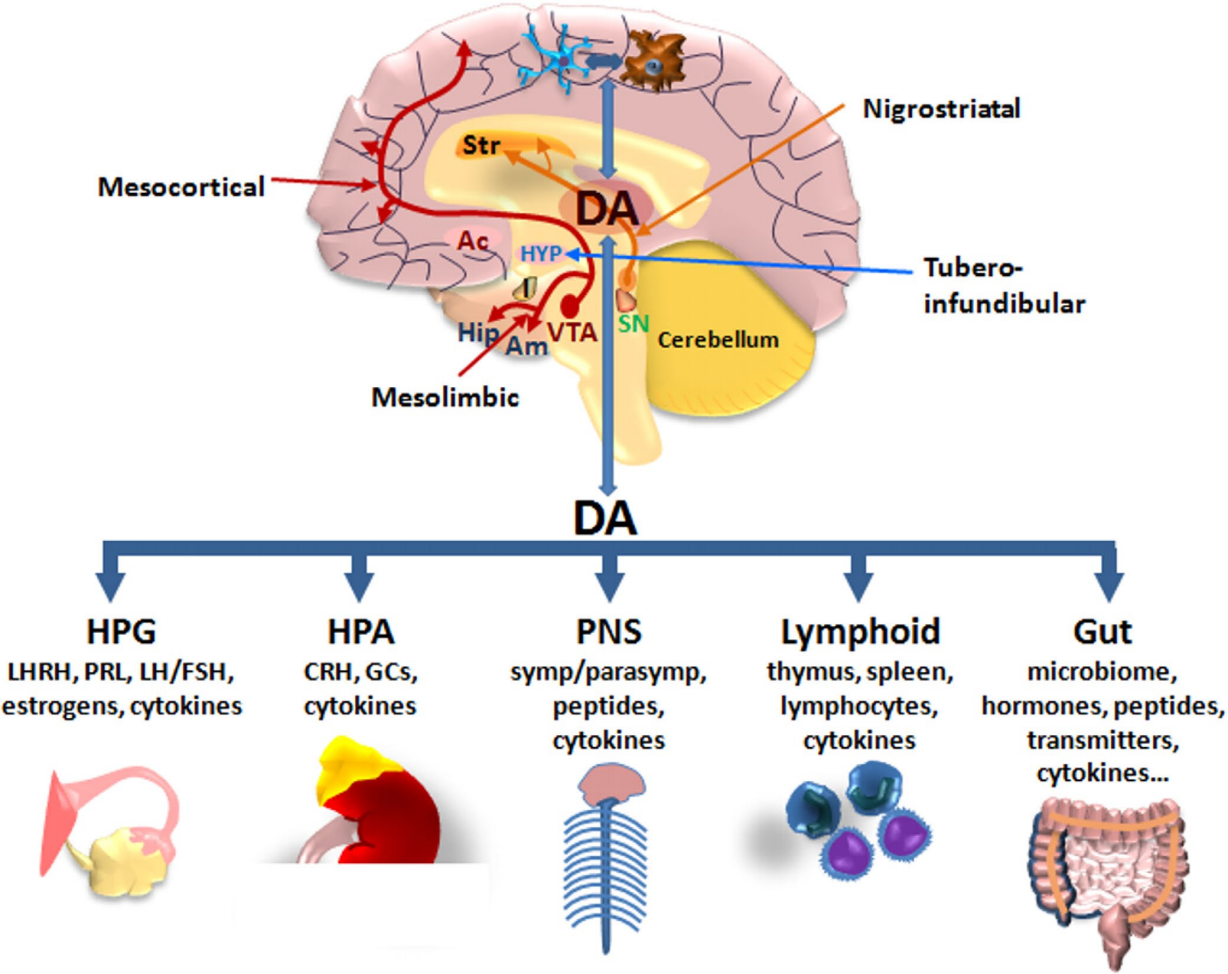
Dopamine as a neuroendocrine–immunomodulator. Schematic representation of DA pathways in CNS and bidirectional DA crosstalk at central and peripheral levels orchestrating the regulation of neuroendocrine, autonomic, lymphoid, and gut axes. Bidirectional circuits linking brain DA to astrocyte and microglial crosstalk are schematically represented. There are three major DA pathways in the brain. The nigrostriatal DA pathway originating in the substantia nigra pars compacta (SNpc, A9) releases DA into the corpus striatum (Str), which governs motor coordination. The mesocortical and mesolimbic DA pathways arise from the ventral tegmental area (VTA, A10), releasing DA into major brain limbic regions, including the nucleus accumbens (Ac), the amygdala (Am), the hippocampus (Hip), and the prefrontal cortex, constituting the mesolimbic–mesocortical reward pathway. Within the hypothalamus (HYP), the tuberoinfundibular DA system modulates the output of releasing factors regulating the hypothalamic–hypophyseal–gonadal (HPG) and hypothalamic–hypophyseal–adrenocortical (HPA) axes, neuropeptides, and hormones, including luteinizing hormone‐releasing hormone (LHRH) and prolactin (PRL), in turn involved in immunomodulation. At peripheral level, DA can communicate with the immune system to modulate its activity, directly through specific receptors in immune organs and cells or indirectly through the peripheral nervous system (PNS), via sympathetic and parasympathetic innervation, neuropeptides, and hormone release. Bidirectional DA crosstalk between CNS and gastrointestinal DA, within the brain–gut axis, also plays roles in modulating microenvironmental cues, including the inflammatory milieu and microbiome homeostasis

Because DA has a multifunctional role as neurotransmitter and neuroendocrine–immune modulator, along with SNpc‐mDAns, other neural populations of the central (CNS) and peripheral nervous systems (PNS) are affected in PD (Braak et al., 2004; Garrido‐Gil et al., 2018; Ulusoy et al., 2017). Aside the DA nigrostriatal pathway, controlling motor coordination, in the ventral tegmental area (VTA, A10), DA‐containing cell bodies release DA into major brain limbic regions including the nucleus accumbens, the amygdala, the hippocampus, and the prefrontal cortex, constituting the mesolimbic–mesocortical reward pathway (Klein et al., 2019) (Figure 1). Within the arcuate nuclei of the mediobasal hypothalamus, the so‐called “tuberoinfundibular DA (TIDA)” system modulates the output of releasing factors within the hypothalamic median eminence (ME), thereby regulating neuroendocrine axes, such as the hypothalamic–hypophyseal–gonadal (HPG) and hypothalamic–hypophyseal–adrenocortical (HPA) axes, neurotransmitters, neuropeptides, and hormones, including luteinizing hormone‐releasing hormone (LHRH) and prolactin (PRL), in turn pivotally involved in immunomodulation (Hodo et al., 2020; Illiano et al., 2020; Maatouk et al., 2019; Marchetti et al., 1990, 2001; Morale et al., 2004; Sarkar et al., 2010) (Figure 1). Accordingly, thanks to the expression of neurotransmitter, peptidergic, hormonal, and cytokine regulatory receptors, glia acts as a preferential neuroendocrine–immune DA target, with DA signaling pathways in turn counter‐modulating inflammatory processes, both at central and at peripheral levels (Figure 1). Importantly, DA contributes to bidirectional neuroendocrine–immune crosstalk, also within the brain–gut axis, with critical implications for PD (Chow & Gulbransen, 2017; Garrido‐Gil et al., 2018; Sampson et al., 2016). Remarkably, emerging functions are being also increasingly reported for the renin–angiotensin system in the regulation of central and peripheral inflammation, collaborating in the complex integration of immune responses (Dang et al., 2021; Gong et al., 2019; Mowry & Biancardi, 2019).

Not surprisingly, within this frame, alterations in a number of non‐motor (including, autonomic, gastric, hormonal, and cognitive) symptoms may both precede and accompany PD onset and progression (Chen, Burton, et al., 2013; Chen, Ni, et al., 2013; Matsumoto, 2015; Tibar et al., 2018).

A number of genes that cause certain forms of inherited PD (<10% cases) have been identified, but the majority of cases (>90%) appear to be sporadic and likely represent an interplay between genetic and environmental influences, with the aging process and inflammation, as main players both in the brain and in the periphery (Bae et al., 2018; Campos‐Acuña et al., 2019; Cannon & Greenamyre, 2013; Di Monte, 2003; Duffy et al., 2018; Gao & Hong, 2011; Gao et al., 2011; Harms et al., 2021; Langston, 2017; Marchetti & Abbracchio, 2005; Tansey & Romero‐Ramos, 2019; Vance et al., 2010). Notably, multiple lines of evidence suggest an interactive network between innate immune cells and the integrity and function of mitochondria, the key organelles maintaining homeostatic cellular balance, critically involved in mDAn health (Schapira et al., 1990, 2014; Vizioli et al., 2020). A compelling link between glial physiopathology and PD genes has been the identification of a panel of mutated genes, including *α*‐synuclein (*SNCA)*, parkin (*PRKN)*, *PINK1*, PTEN‐induced putative kinase *(DJ1)*, and leucine‐rich repeat kinase 2 (*LRRK2)* in astrocytes and/or microglial cells (Ashley et al., 2016; Barodia et al., 2019; Booth et al., 2017; Choi et al., 2016; Dzamko et al., 2015). Particularly, the pathways regulated by these genes intersect DA signaling and mDAn health at the interface of key cellular functions affected in both aging and Parkinson's disease, namely, the inflammatory response, endoplasmic reticulum (ER) stress, and mitochondrial, lysosomal, proteasomal, autophagic, and *Wingless*‐*type mouse mammary tumor virus integration site (Wnt)*/*β*‐*catenin* signaling functions (Arias, 2017; Awad et al., 2017; Bektas et al., 2019; Belenkaya et al., 2008; Berwick & Harvey, 2012, 2014; Berwick et al., 2017; Cuervo, 2008; Cuervo & Macian, 2014; Kim et al., 2013; Marchetti, 2018; Schmidt et al., 2011). On the contrary, potential neuroprotective and neuroreparative functions of astrocytes and microglia are being increasingly reported, thereby supporting the initial claim “To be or not to be inflamed: is that the question in anti‐inflammatory drug therapy of neurodegenerative diseases?” (Marchetti & Abbracchio, 2005), underscoring “*Dr Jekyll*/*Mr Hyde*” sides of glia, yet the crucial mechanisms/conditions driving a “beneficial glial switch,” whereby astrocytes and microglia can exert neuroprotective and/or proregenerative properties upon injury, remain ill‐defined.

One critical feature of astrocytes is to protect the vulnerable mDAns. Research of the last decade from our laboratory centered on *Nuclear factor erythroid 2*‐*like 2* (*NFE2L2*/Nrf2), the master regulator of cellular defense against oxidative stress and inflammation, and a critical modulator of the life span (Ammal Kaidery et al., 2019; Cuadrado et al., 2019; Dinkova‐Kostova & Abramov, 2015; Holmström et al., 2016; Johnson & Johnson, 2015; Lastres‐Becker, 2021; Ryoo & Kwak, 2018; Strong et al., 2016), and the Wnt/β‐catenin signaling cascade, a vital pathway for mDAn neurogenesis, neuroprotection, and immunomodulation, and key interactor of the aging process (Arias, 2017; Awad et al., 2017; Berwick & Harvey, 2012, 2014; Berwick et al., 2017; Galli et al., 2014; Hofmann et al., 2014; Harvey & Marchetti, 2014; Knotek et al., 2020; L’Episcopo, Tirolo, et al., 2011; L’Episcopo, Serapide, et al., 2011; L’Episcopo, et al., 2013; Marchetti & Pluchino, 2013; Marchetti et al., 2020).

Notably, it should be emphasized that being a critical neuropathological hallmark of aging and aging‐dependent diseases, especially PD, inflammatory response regulation is multifaceted and integrated by a wide panel of crucial intermingled pathways to include, besides others, the renin–angiotensin system and a wide panel of neurotransmitters, and hormonal and peripheral immunoregulatory networks, recently summarized in excellent reviews and original contributions (Dang et al., 2021; Hodo et al., 2020).

Considering the complexity of the mutual interplay of glial‐derived factors *in vivo*, coupled to the influence of different risk factors in mDAn vulnerability, it is conceivable that DA signaling at the astrocyte–microglial interface will have a prominent impact for mDAn survival and health, especially in light of the intrinsic characteristics of mDAns, the interplay between DA signaling mechanisms, coupled to the region‐specific properties of nigrostriatal glial cells (Asanuma et al., 2019; Kostuk et al., 2019; Sofroniew, 2015; Wang et al., 2020; Yao et al., 2021).

With a major focus on DA intersection within the astrocyte–microglial inflammatory network in PD vulnerability with age, we herein first summarize the characteristics of DA receptor signaling systems, the propensity of DA neurons to oxidative stress/glial inflammatory triggers dictating the vulnerability to PD. Reciprocally, DA modulation of astrocytes and microglial reactivity, coupled to the convergent impact of gene–environment interactions, then constitute a further level of control impacting on mDAn survival/death. Not surprisingly, within this circuitry, DA acting as a neuroendocrine–immune modulator converges to modulate the *Nrf2*/*Wnt* signalosome, adding to the already complex “signaling puzzle,” a novel actor in mDAn–glial regulatory machinery. Here, an autoregulatory feedback system is proposed allowing DA to act as an endogenous *Nrf2*/*Wnt* innate modulator, thereby linking DA‐induced oxidative stress to most important neuroprotective pathways in PD, then tracing the importance of DA receptor agonists applied to the clinic as immune modifiers.

### 1.1 DA receptor signaling and oxidative stress: a unique link for mDAn vulnerability in Parkinson's disease

**FIGURE 2:**
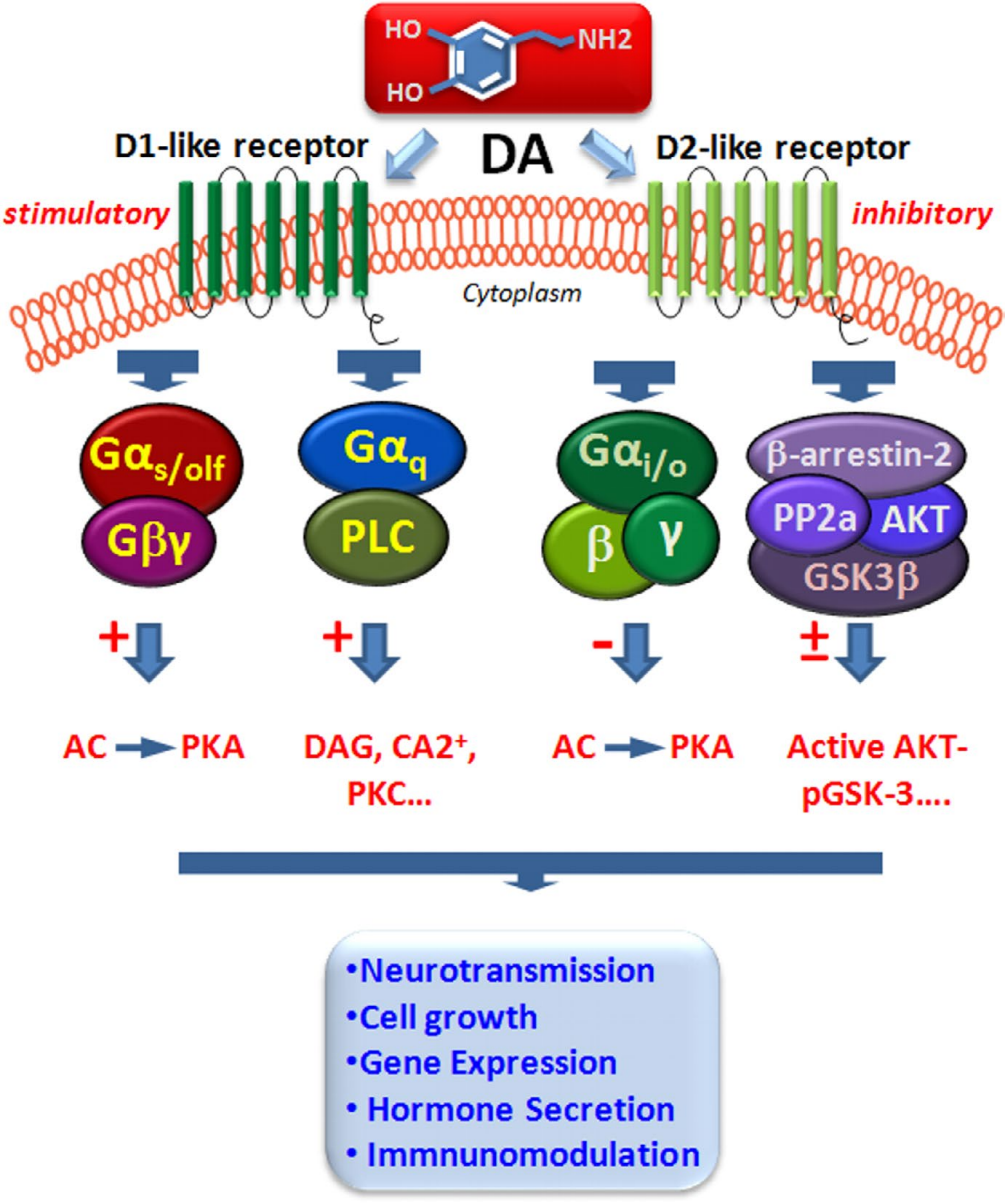
Dopamine receptors and signaling pathways in neuroimmune network. Simplified schematic representation of DA acting via DRD1‐ and DRD2‐like receptors by G protein‐dependent, by stimulatory (Gαs) or inhibitory Gαi/o subunits, or by G protein‐independent β‐arrestin‐2 (βArr2)‐dependent pathway (for details, see the text). DA binding to DRD1‐like receptor subtypes can elicit two transduction pathways, of which one is coupled to Gαs/olf, driving adenylyl cyclase increasing cyclic adenosine monophosphate (cAMP) activity. In addition to DRD1 effects on cAMP‐regulated signaling, DRD1Rs couple to Gαq to modulate phospholipase C (PLC) pathway, in turn activating phospholipid turnover and increasing diacylglycerol (DAG), releasing Ca2+ from internal stores, and activating protein kinase C (PKC). D2‐like receptor subtypes, coupled to Gαi/o, suppress cAMP activity, thereby producing an inhibitory effect upon DA binding. The G protein‐independent D2R signaling is represented by βArr2‐mediated signaling. The activation of the D2‐like receptors contributes to the constitution of a protein complex composed of protein phosphatase 2A (PP2A), serine/threonine kinase (Akt), and βArr2, where PP2A increases the dephosphorylation and inactivation of Akt, leading to the modulation of glycogen synthase kinase‐3 (GSK‐3) activation

Five subtypes known as “D1‐like (DRD1 and DRD5) and “D2‐like” (DRD3 and DRD4) receptors, belonging to the superfamily of G protein‐coupled receptors (GPCRs), mediate all physiological functions of DA, as expanded in comprehensive reviews of the field (Beaulieu et al., 2015; Beaulieu & Gainetdinov, 2011; Gurevich et al., 2016). Upon DA binding, DRD1‐like receptor subtypes, coupled to Gαs/olf, drive adenylyl cyclase and thus cyclic adenosine monophosphate (cAMP) activity, then promoting cAMP‐dependent protein kinase A activation engendering phosphorylating cascades (Figure 2). In addition to DRD1 effects on cAMP‐regulated signaling and Src family kinase (SFK) pathway, DRD1/DRD2 heterodimers or DRD5 can couple to Gαq to modulate phospholipase C (PLC), in turn activating phospholipid turnover and diacylglycerol (DAG), releasing Ca^2+^ from internal stores, and activating protein kinase C (PKC) (Figure 2).

On the contrary, D2‐like receptor subtypes, coupled to Gαi/o, suppress cAMP activity, thereby producing an inhibitory effect upon DA binding. In addition, DRD2‐mediated activation of Gβγ subunits also participates in the modulation of ion channels, including G protein‐coupled inwardly rectifying potassium channels (GIRKs) and L‐type calcium channels (Beaulieu et al., 2015; Beaulieu & Gainetdinov, 2011). The G protein‐independent DRD2 signaling is represented by β‐arrestin‐2 (βArr2)‐mediated signaling (Figure 2). The mechanism underlying the regulation of Akt by βArr2 has shown that activation of the D2‐like receptors contributes to the constitution of a protein complex composed of protein phosphatase 2A (PP2A), Akt, and βArr2 involved in a panel of intermingled signaling pathways (reviewed by Beaulieu et al., 2015), including the modulation of glycogen synthase kinase‐3 (GSK‐3), a multifunctional enzyme intersecting a wide variety of survival and immunomodulatory pathways (see Beurel et al., 2015, and details in next sections) (Figure 2).

#### 1.1.1 Aging, dopamine transporter (DAT), and the vulnerability to PD

**FIGURE 3:**
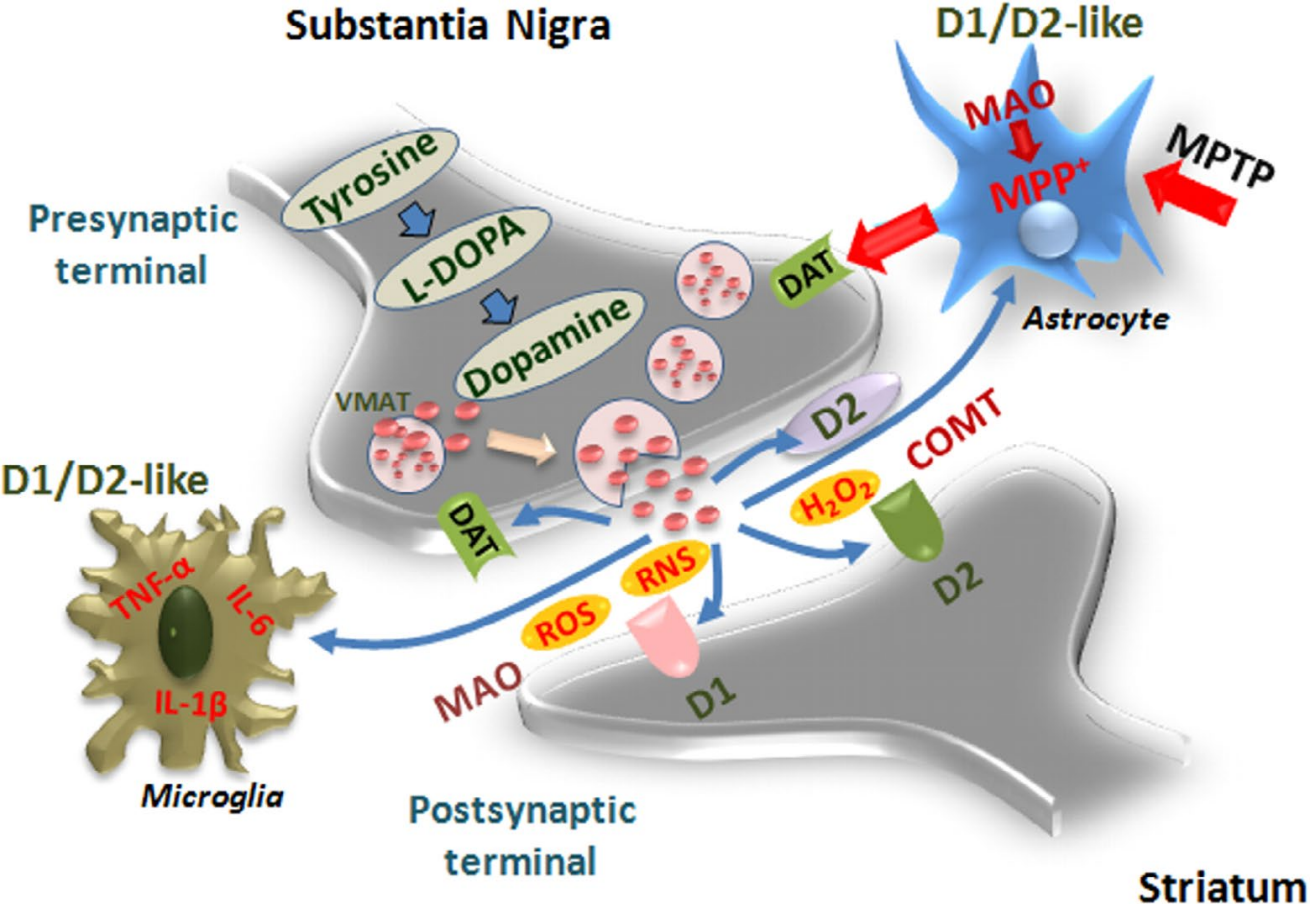
Dopamine metabolic pathways and astrocyte–microglial oxidative/inflammatory network. A schematic view of DA pre/postsynaptic regulatory functions. DA biosynthetic steps start with the action of the enzyme tyrosine hydroxylase (TH), the rate‐limiting step in the biosynthesis of DA in the presynaptic terminals to form the DA precursor, L‐DOPA, the principal drug in the therapeutic management of PD. Next, L‐DOPA is decarboxylated to form DA. DA is next incorporated into synaptic vesicles, via the vesicular monoamine transporter 2 (VMAT2), permitting its protection from metabolic inactivation, and its storage until stimulation, when DA released by exocytosis then reaches postsynaptic neurons and binds to cognate D1‐ and D2‐like receptors. D2 presynaptic (inhibitory) receptor can stop the further production and release of DA. The reuptake of DA by presynaptic terminals through the actions of the high‐affinity DA transporter (DT) represents another key step whereby DA is recycled back into the storage vesicles, responsible for modulating the concentration of extraneuronal DA in the brain. Two enzymes are responsible for DA inactivation, monoamine oxidases (MAOs) and catechol‐*O*‐methyl transferase (COMT), predominantly expressed by astrocytes. During DA metabolic steps, reactive oxygen (ROS) and nitrogen (RNS) species can be produced, which may further engender a neurotoxic cycle capable of causing cell death (for details, see the text). Astrocyte–neuron dialogue may be harmful upon exposure to 1‐methyl‐4‐phenyl‐1,2,3,6‐ tetrahydropyridine (MPTP), as the neurotoxin is converted to its active metabolite in astrocytes, MPP^+^, then specifically transported by DAT and concentrated within the nigral DA neurons where it inhibits complex I of the mitochondrial electron transport chain, resulting in ATP depletion and subsequent neuronal cell death. This process associated with a robust microgliosis and proinflammatory cytokines, tumor necrosis factor α (TNF‐α), and interleukin‐1β (IL‐1 β) production can be counter‐modulated by DA anti‐inflammatory effects via D1/D2‐like receptors in glial cells, as discussed in Sections 1.3–1.5

In the presynaptic terminal, the reuptake of DA through the actions of the high‐affinity DA transporter (DAT) represents a key step whereby DA is repackaged into the storage vesicles by the action of the vesicular monoamine transporter, VMAT. DAT is a sodium‐coupled symporter protein belonging to the superfamily of SLC transporters, responsible for modulating the concentration of extraneuronal DA in the brain (Amara & Kuhar, 1993). Notably, association of a polymorphism in the DAT gene with Parkinson's disease (Le Couteur et al., 1997; Wang et al., 2000) underlines its potential role in PD vulnerability (Schmitt et al., 2013). Specifically, age‐dependent changes in DAT and accumulation of nitrosylated tyrosine (3‐nitrotyrosine, 3‐NT) in rhesus monkey (Kanaan et al., 2008) and rodent mDAns (Marchetti et al., 2013) support dysfunctional DAT as a vulnerability factor for nigrostriatal degeneration. Particularly, recent studies of Illiano et al. (2020) showed that in rodents, the lack of DAT results in increased vulnerability and aberrant autonomic response to acute stress. In particular, DAT represents a preferential target for parkinsonian neurotoxins, as the active metabolite of 1‐methyl‐4‐phenyl‐1,2,3,6‐tetrahydropyridine (MPTP), MPP^+^, is specifically transported by DAT and concentrated within the nigral DA neurons where it inhibits complex I of the mitochondrial electron transport chain (METC), resulting in ATP depletion and subsequent neuronal cell death (Di Monte & Langston, 1995; Langston, 2017; Schildknecht et al., 2017) (Figure 3). The induction of oxidative stress results in the opening of mitochondrial permeability transition pore (mPTP), the release of cytochrome C, and the activation of caspases. It seems important to recall that mitochondria represent the primary energy‐generating system, involved in multiple processes, including energy metabolism, reactive oxygen (ROS) generation, mitochondrial dynamics, and distribution (Blesa et al., 2015; Bose & Beal, 2016; Schildknecht et al., 2017). Of specific mention, mitochondrial damage due to Ca^2+^ overload‐induced opening of mPTP is believed to play a key role in selective degeneration of nigrostriatal DAns in PD. Hence, endoplasmic reticulum (ER) acts as a reservoir of Ca^2+^ ions, and increased Ca^2+^ released from the ER further enhances mitochondrial oxidative stress of mDAns in SNpc (Blesa et al., 2015; Schildknecht et al., 2017). Reportedly, reduction in complex I activity in the SNpc of patients with sporadic PD has been well described, being considered as one of the primary sources of ROS in PD, and accounting for the majority of mDAn cell death (Hattori et al., 1991; Hattingen et al., 2009; Schapira et al., 1990). Of note, in Str, DA terminals actively degenerated proportionally to increased levels of DA oxidation following a single injection of DA into the striatum (Rabinovic et al., 2000).

Not only too little but also too much of DAT‐mediated mechanisms may have harmful consequences, since increased uptake of DA through DAT in transgenic (Tg) mice overexpressing the DA transporter results in oxidative damage, neuronal loss, and motor deficits (Masoud et al., 2015). Here, the effects of increased DAT expression on DA homeostasis, neuronal survival, oxidative stress, and motor behavior of DAT‐Tg mice were evaluated together with the nigrostriatal response to MPTP (Masoud et al., 2015). Hence, an almost 30%–36% loss of mDAns and fine motor deficits were associated with an increased vulnerability to MPTP‐induced mDAn loss, indicating that overactivation of DAT‐mediated uptake of dopamine leads to basal neurotoxicity and heightened sensitivity to exogenous insults (Masoud et al., 2015).

Together, the presynaptic transporter DAT in nigral dopaminergic neurons confers susceptibility and represents a principal age‐dependent vulnerability factor for PD.

#### 1.1.2 Aging, DA oxidative metabolism, and nigrostriatal neuron vulnerability in PD

Given the high metabolic activity that is required to support their extensive axonal arborization, mDAns are physiologically subjected to various levels of oxidative stress, and reciprocally, among a number of brain regions studied, the SNpc, where A9 DA cell bodies are located, is the more vulnerable region, as DA metabolism constantly generates ROS (Chinta & Andersen, 2008). Notably, the aging process, associated with a progressive mDAn dysfunction, may add a further oxidative load to the system, with harmful consequences for nigrostriatal neuron integrity (as summarized in Section 1.2). Two enzymes are primarily responsible for DA inactivation, monoamine oxidase isoforms (MAO‐A and MAO‐B) and catechol‐*O*‐methyl transferase (COMT), predominantly expressed by glial cells. MAO, a flavin‐containing enzyme is located on the outer membrane of the mitochondria. This enzyme oxidatively deaminates catecholamines to their corresponding aldehydes; these can be in turn converted either by aldehyde dehydrogenase to acids or by aldehyde reductase to form glycols. Due to its intracellular localization, MAO has a strategic role in the inactivation of DA when the amine is not protected by the storage vesicles in presynaptic terminal. MAO breaks down DA to 3,4‐dihydroxyphenylacetaldehyde (DOPAL), which, in turn, is degraded to form 3,4‐dihydroxyphenylacetic acid (DOPAC) by the action of the enzyme aldehyde dehydrogenase. COMT converts DA to 3‐methoxytyramine (3‐MT), which is further reduced by MAO to homovanillic acid (HVA) and then eliminated in the urine. During DA metabolic steps, ROS and RNS can be produced (Afanas, 2005). These may include hydrogen peroxide (H_2_O_2_), singlet oxygen (^1^O2), hydroxyl (OH), and superoxide (O2) radicals (Halliwell & Gutteridge, 1984; Kumar et al., 2012; Sies et al., 2017). RNS are produced in neuronal cells from arginine by the neuronal nitric oxide synthase (nNOS) and include nitric oxide (NO), nitrite (NO2), and S‐nitrosothiols and peroxynitrite (OONO) (Adams et al., 2015). Additionally, DA metabolites and certain derivatives such as N‐methyl‐(R)‐salsolinol (NMSAL) (Naoi et al., 2002) are prone to oxidation, generating reactive quinones, which may further engender a neurotoxic cycle able to readily modify proteins and potentially cause protein aggregation (Sulzer & Zecca, 2000; Zucca et al., 2014).

Overall, age‐ and PD‐dependent chronic DA neuronal dysfunction, altered DA metabolism, and dysregulated reactive species production then have to face the harmful gene x environment interactions promoting a feedforward oxidative/inflammatory cycle, contributing to progressive neuronal deterioration and motor deficiency of PD.

### 1.2 The vicious cycle of aging, genes, and mDAn mitochondrial dysfunction

#### 1.2.1 Aging and the glial inflammatory network in PD

A critical hallmark of aging is the progressive decline in nigrostriatal DA neurons (Bezard & Gross, 1998; Boger et al., 2010; Collier et al., 2007; de la Fuente‐Fernández et al., 2011; Hindle, 2010) associated with the failure of the adaptive/compensatory potential of mDAns, recognized to be implicated in the slow but progressive nigrostriatal degeneration of PD, with the late appearance of clinical signs (Bezard & Gross, 1998; Hornykiewicz, 1993; Kanaan et al., 2008). Here, a crucial causative role is represented by the exacerbation of the astroglial microenvironment, as a result of a dysfunctional gene–environment crosstalk. Reportedly, the major aging culprits, namely oxidative stress and low‐grade inflammation, may further be exacerbated under basal ganglia injury, neurotoxin exposure, male gender, and PD genetic mutations (Gao & Hong, 2011; Gao et al., 2003, 2011; Hu et al., 2008; Marchetti & Abbracchio, 2005). Notably, with age, defective mitochondrial turnover by autophagy may trigger chronic inflammation and critically contribute to the impairment of immune defense, in as much as malfunctioning autophagy has been reported in several diseases NDs, including PD, with its consequent toxicity considered to be a main cause of the disease (Bektas et al., 2019; Cuervo, 2008; Cuervo & Macian, 2014; Scrivo et al., 2018). Recently, the pentose phosphate pathway (PPP, a metabolic pathway parallel to glycolysis), which converts glucose‐6‐phosphate into pentoses and generates ribose‐5‐phosphate and NADPH thereby governing anabolic biosynthesis and redox homeostasis, has gained a critical attention (Tu et al., 2019). Hence, expression and activity of G6PD were elevated in an in vitro model of PD (e.g., LPS‐treated midbrain neuron–glial cultures) and the SN of vivo PD models, associating with microglial activation and mDAn neurodegeneration, whereas inhibition of G6PD elevation or knockdown of microglial G6PD attenuated LPS‐elicited chronic mDAn neurodegeneration (Tu et al., 2019). Further, microglia with elevated G6PD activity/expression produced excessive NADPH and provided abundant substrate to overactivated NADPH oxidase (NOX2) resulting in exacerbated ROS, which suggests that G6PD and NOX2 are potential therapeutic targets for PD (Tu et al., 2019).

In glial cells, mutated genes then cooperate with environmental influences to impair mitochondrial homeostasis and the autophagy–lysosomal pathways, all implicated in mDAn dysfunction observed in PD (Ashley et al., 2016; Barodia et al., 2019; Booth et al., 2017; Choi et al., 2016; Cuervo & Macian, 2014; Dzamko et al., 2015; Gillardon et al., 2012; Kim et al., 2013; Kline et al., 2021; Lastres‐Becker et al., 2012; Marchetti, 2020; Schmidt et al., 2011). While this topic is outside the scope of the present work, it seems important to underscore that all major human PD‐linked mutations (i.e., *SNCA*, *LRRK2*, *PINK1*, and *DJ1)* induce complex I inhibition, and synergism with the age‐dependent oxidative stress and inflammation further promotes increased generation of oxidative and nitrosative stress mediators, in turn exacerbating the proinflammatory microglial “M1” phenotype, then promoting progression of mDAn death (Lastres‐Becker et al., 2012). Notably, with age, progressive acquisition by glial cells of the capacity to produce greater levels of a set of proinflammatory mediators both in physiological conditions, and more actively under immune or neurotoxic stimuli on the one hand, coupled to the failure of host surveillance systems, on the other, can translate into harmful consequences both at central and at peripheral levels (Boche et al., 2013; De Cecco et al., 2019; Perry & Teeling, 2013; Tansey & Romero‐Ramos, 2019). This so‐called “microglial cell shift” to the “harmful,” M1 phenotype promoting the release of an array of factors that are detrimental for the vulnerable mDAns depends upon inflammasome activation.

#### 1.2.2 Aging, inflammasome activation, and mitochondrial dysfunction in PD

Significantly, nuclear factor kappa‐light‐chain‐enhancer of activated B cells (NF‐ĸB, a protein complex that controls cytokine production and cell survival) is the first signal for inflammasome induction and a key interactor of DA signaling (as detailed in the next section). Among the numerous inflammatory cytokines, interleukin‐1β (IL‐1β) produced by glial cell Nod‐like receptor protein (NLRP) inflammasome exerts a central role in regulating neuroinflammation (Codolo et al., 2013; Haque et al., 2020; Heneka et al., 2014). Upon stimulation by adenosine triphosphate (ATP), reactive oxygen species, lysosomal contents, or other factors, NLRP3 recruits the adapter molecule apoptosis‐related speck‐like protein (ASC) and procaspase‐1 to promote caspase‐1 activation (Dinarello, 2007). This process leads to the maturation of the proinflammatory cytokines (IL‐1β, IL‐18). The secretion of IL‐1β by glial cells contributes toward the destruction of mDAns in the brain of PD patients and the initiation of cell death (McGeer & McGeer, 2008). Hence, MPTP‐driven NLRP3 inflammasome activation in microglia plays a central role in mDAns demise (Gordon et al., 2018; Lee, 2018), in as much as aging represents a synergic trigger directing microglia toward the M1 proinflammatory phenotype (L’Episcopo, Tirolo, Testa, Canigilia, Morale, Impagnatiello, et al., 2011). Additionally, mitochondrial impairment in microglia amplifies NLRP3 inflammasome proinflammatory signaling in cell culture and animal models of PD (Sarkar et al., 2017; Zhu et al., 2021), whereas the suppression of NLRP3 inflammasome‐derived proinflammatory cytokines mitigates mDAn degeneration and may be beneficial to PD patients (Ahmed et al., 2021; Gordon et al., 2018; Haque et al., 2020; Zhu et al., 2018). Interestingly, the vicious crosstalk between the impaired mitochondrial signaling and NLRP3 machinery can contribute to amplify further the noxious mDAns outcome, as NLRP3/caspase‐1 activation under toxic exposure is mediated by mitochondrial ROS generation (Afonina et al., 2017; Sarkar et al., 2017).

Altogether, glia acts as a common final pathway of gene x environment interactions in PD, playing critical roles in the exacerbation of age‐dependent mDAn degeneration, and intersecting the harmful DA oxidative metabolism. As a result, the modulatory role of DA signaling in glial cell networks appears decisive, since they might either help the imperiled mDAns to combat oxidative stress and inflammation through a wide variety of mechanisms addressed in the following sections.

### 1.3 Dopamine signaling strategy to combat oxidative stress and inflammation in PD

Indeed, within this scenario, DA emerges as a pivotal regulator of inflammation, thanks to its dual facet of immunosuppressor/activator relying on its receptor subtypes coupled to stimulatory/inhibitory signal transduction pathways. Reportedly, exposure to DA or DA receptor agonists decreases detrimental actions of immune cells (Table 1). In contrast, a reduction in DA signaling perpetuates a proinflammatory state associated with increased release of proinflammatory molecules. Here, DA dialogue with microglia and astrocytes together with the proposed DA‐mediated intersection at the *Nrf2*/*Wnt*/*β*‐*catenin*/*GSK*‐*3β* signalosome is presented.

**TABLE 1:** Dopamine signaling at the microglial‐astrocyte interface

Da receptor subtypes and functions	References
**ASTROCYTES AND MICROGLIA**	
Cultured human elderly microglia expressed mRNAs for DRD1‐D4 but not DRD5. The microglia, as well as PD microglia *in situ*, were also immunoreactive for DRD1‐D4 but not DRD5.	Mastroeni et al. (2009)
DA has a differential role in resting and activated microglia, as phagocytosis and adhesion depend on the activation states of microglia.	Fan et al. (2018)
DA prevents microglial glutamate release evoked by α‐synuclein aggregates by an antioxidant effect requiring DRD1 activation and PI3K inhibition.	Dos‐Santos‐Pereira et al. (2018)
In PD rats DRD1 activated by acetyl‐L‐carnitine attenuates microglial activation and the release of pro‐inflammatory mediators, preventing neuronal death and improving memory functions.	Singh et al. (2018a)
In ageing mice, progressive decline of DA‐activating DR2R associates with increased reactivity of microglia, further amplified after basal ganglia injury, in the face of downregulation of major DRD2, anti‐oxidant and neuroprotective astrocyte transcripts and protein levels.	L’Episcopo et al. (2018) Serapide et al (2020)
DRD1 are present on fine processes of GFAP^+^ astrocytes in the substantia nigra pars reticulata being a major candidate to receive DA released dendritically.	Nagatomo et al. (2017)
DRD3 are selectively expressed in astrocytes but not in microglia. DRD3 selective antagonist PG01037 reduces the acquisition and activation of M1 microglia, and contributes to anti‐inflammatory effects, with therapeutic effects in PD mice model. DRD3 deficiency resulted in exacerbated expression of the anti‐inflammatory protein “found in inflammatory zone 1” (Fizz1) in glial cells both *in vitro* and *in vivo*.	Elgueta et al (2017) Montoya et al. (2019)
DRD2 agonists suppress the upregulation of caspase‐1 and IL‐1β expression in primary cultured mouse astrocytes in response to LPS plus ATP‐induced NLRP3 inflammasome activation. Astrocyte DRD2 receptor restricts astrocytic NLRP3 inflammasome activation via enhancing the interaction of βArr2 and NLRP3.	Zhu et al. (2018)
DRD2 agonists significantly mitigate LPS‐induced inflammatory response in astrocytes, while α‐Syn disrupts the anti‐inflammatory role of DRD2 interfering with β‐arrestin2‐TAB1 interaction in astrocytes.	Du et al. (2018)
DA downregulates astrocyte‐derived angiotensin I and regulates microglial angiotensin receptors, with inhibition of proinflammatory microglia phenotype under LPS activation.	Dominguez‐Meijide et al. (2017)
There is an inverse relationship between microglia inflammatory activation and the sharp inhibition of DA, DRD2 and DAT in striatum during ageing and basal ganglia‐injury. Robust activation of major proinflammatory transcripts, including Nfkb, IL‐1α, TNF‐α, and IL‐6, as well as oxidative and nitrosative stress markers such as ROS, RNS, and 3‐NT coinciding maximal glial activation.	L’Episcopo et al (2012; 2013)
Astrocytic DA modulation carried out by DRD2 can suppress neuroinflammation through CRYAB‐ dependent mechanism, whereas DRD2 knockout mice showed robust inflammatory responses and increased vulnerability of mDAns to MPTP.	Shao et al. (2013)
DRD2 receptor activation by Sinomenine in astrocytes alleviates neuroinflammatory injury via the CRYAB/STAT3 pathway.	Qiu et al (2016)

Abbreviations: 3‐NT, 3‐nitrotyrosine; CRYAB, α‐beta‐cristallin; DA, dopamine; DRD1‐DRD5, dopamine receptor 1‐5; Fizz1, found in inflammatory zone 1; IL‐1 β, interleukin‐1β; IL‐6, interleukin‐6; iNOS, inducible nitric oxide; NF‐ĸB, nuclear factor kappa‐light‐chain‐enhancer of activated B cells; NLRP3, Nod‐like receptor protein 3; reactive nitrogen species, RNS; reactive oxygen species, ROS; TAB1, transforming growth factor beta 1; TNF‐α, tumor necrosis factor α; α‐Syn, α‐Synuclein; βArr2, β‐Arrestin 2.

#### 1.3.1 DA signaling intersects harmful microglial inflammatory networks in PD: DA/NF‐ĸB /NLRP3 crosstalk

A most robust evidence linking DA to inflammation is the recognized notion that DA deficit within the nigrostriatal system, as observed in preclinical and clinical models of PD, strongly associates with exaggerated inflammation both at central and at peripheral levels. Studies conducted in the MPTP model of PD, including our own results, clearly showed an inverse relationship between microglial inflammatory activation and the sharp inhibition of DA, DRD2 and DAT in striatum of basal ganglia‐injured mice (Serapide et al., 2020). The greatest effects were observed in aged mice, coincident with a robust activation of major proinflammatory transcripts, including NF‐ĸB, IL‐1β, TNF‐α, and IL‐6, as well as oxidative and nitrosative stress markers such as ROS, RNS, and 3‐NT. Likewise, at the midbrain level, progressive decline in DA resulting from the aging process associates with increased reactivity of the microglial cell compartment, further amplified after basal ganglia injury, in the face of downregulation of major DA transcripts, including DRD2 and protein levels in the midbrain (L’Episcopo et al., 2018). Such a dramatic loss of DA inhibitory tonus onto nigrostriatal astrocytes and microglia likely contributes to the observed exacerbated neuroinflammation during aging and PD (Serapide et al., 2020).

Reportedly, microglia harbor DA receptor subfamilies (Pocock & Kettenmann, 2007). Studies of Mastroeni et al. (2009) showed that cultured human elderly microglia expressed mRNAs for DRD1‐DRD4 but not DRD5 receptors (Table 1). In addition, PD microglia *in situ* were also immunoreactive for DRD1‐DRD4 but not for DRD5 receptors, suggesting that activated PD microglia expressing DA receptors might play roles in the selective vulnerability of DA neurons in PD (Mastroeni et al., 2009). In PD rats, DRD1 activated by acetyl‐L‐carnitine attenuates microglial activation and the release of proinflammatory mediators, a phenomenon potentially linked to the amelioration of cognitive deficits and neurodegeneration (Singh, Mishra, Mohanbhai, et al., 2018). Here, acetyl‐L‐carnitine inhibited microglial activation‐mediated inflammatory response and weakened TNF‐α levels by increasing the production of the anti‐inflammatory cytokine, IL‐10, which led to improved neuronal survival (Singh, Mishra, Mohanbhai, et al., 2018), implicating DA regulation of inflammasome/NF‐ĸB pathway (recently reviewed by Feng & Lu, 2021). Within this context, emerging evidence also indicates that microglial polarization and generation of ROS are tightly related to the DA‐targeted brain intrinsic renin–angiotensin system (RAS), a local/paracrine modulatory mechanism playing an important role in inflammatory processes (Dang et al., 2021; Dominguez‐Meijide et al., 2017; Gong et al., 2019; Mowry & Biancardi, 2019; Xia et al., 2019).

**FIGURE 4:**
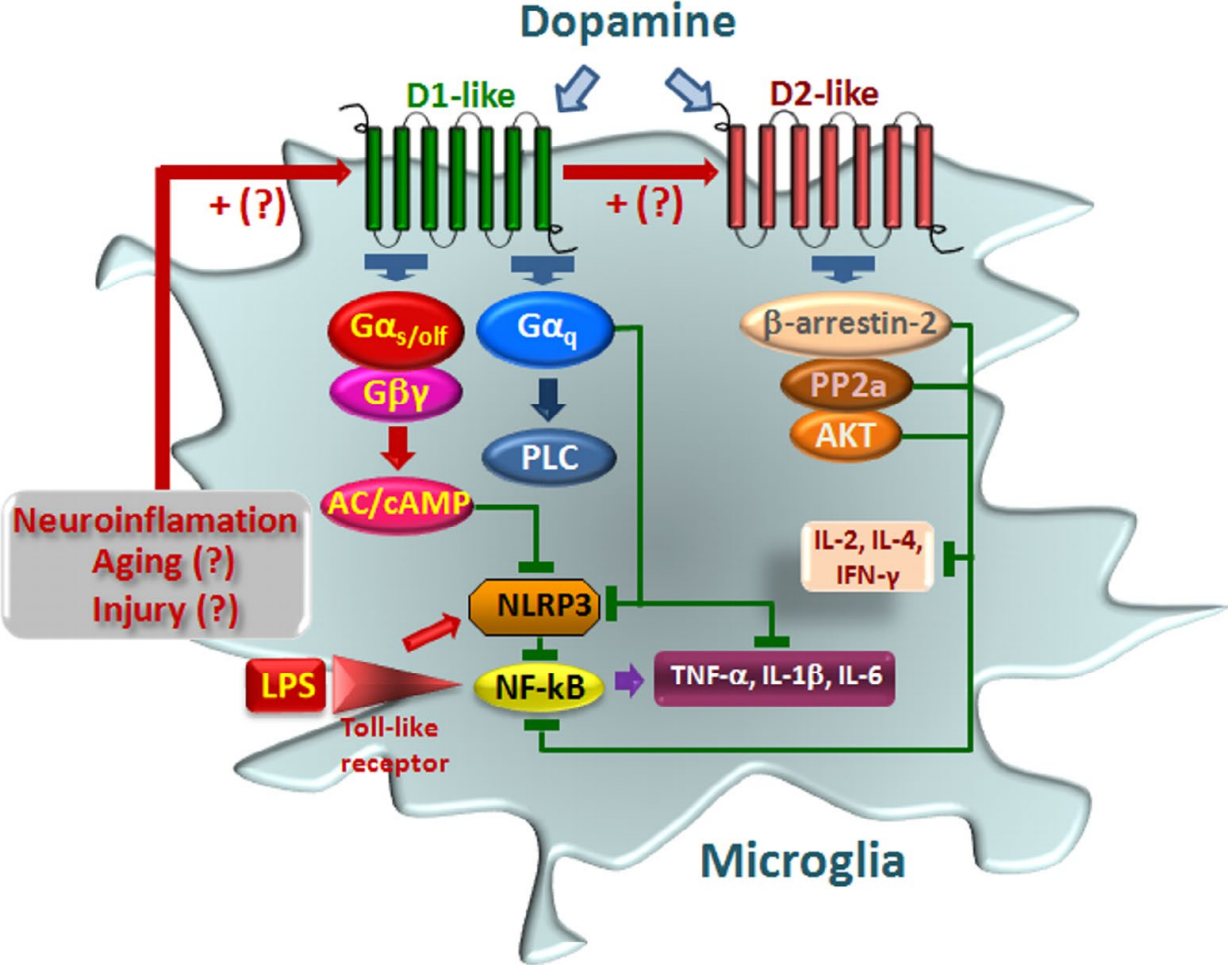
Dopamine signaling pathways modulate inflammasome activation in microglia. Immune activation is schematically represented. LPS via Toll‐like receptors (TLRs) activates Nod‐like receptor protein 3 (NLRP3) inflammasome and nuclear factor kappa‐light‐chain‐enhancer of activated B cell (NF‐ĸB) signaling pathways promoting proinflammatory cytokine (IL‐1β, TNF‐α, IL‐6) release (detailed in Section 1.2). DA and DA agonist activation of D1‐like receptors (D1 and D5) results in a downmodulation of immune response. D1 activation via Gα_solf_ increases cAMP, which binds directly to NLRP3 triggering its ubiquitination via an autophagy‐mediated degradation. Activated cAMP signaling also inhibits p65/RelA and p50 activation. D5R activation directly recruits a multiprotein complex, impairing activation of NF‐kB. Activation of D2R‐b‐arrestin‐2 complex also results in D2R binding to NLRP3 to repress its activation. D2R signaling can negatively regulate the NF‐kB signaling pathway, thereby inhibiting major proinflammatory cytokine release. The hypothetical role of neuroinflammation, aging, and brain injury, as a counter‐regulatory mechanism, via upregulation of DA receptor expression is illustrated

The roles of DA signaling in regulating these key inflammatory pathways stem from the transduction machinery of DRD1/DRD2‐like receptor subtypes. In DRD1‐like receptors, the elevated cAMP induced by DA directly binds to NLRP3 (Figure 4). Here, elevated cAMP activates PKA and phosphorylates cAMP‐response element binding protein (CREB), thus disrupting NF‐ĸB homeostasis and resulting in the inhibition of the inflammatory response (Neumann et al., 1995; Xia et al., 2019 and Refs herein). Also, via DRD5 signaling, DA can block NF‐kB pathway, thus suppressing proinflammatory mediators (Wu et al., 2020; Zhang et al., 2015). DRD2‐like receptor signaling may involve either a *GPC*‐dependent or a *β*‐*arrestin*‐dependent *GPC*‐independent pathway to modulate glial inflammatory activation, with the *β*‐*arrestin*‐dependent mechanism playing a critical role (Fan, 2014) (Figure 4).

Interestingly, DA receptor expression is induced by the activated microglial phenotype, as cerebral ischemia induced the expression of DRD2 on Iba1‐immunoreactive inflammatory cells in the infarct core and penumbra (Huck et al., 2015). Similarly, DA has a differential role in influencing cellular functions of resting and activated microglia, such as phagocytosis and adhesion, depending on the activation states of microglia (Fan et al., 2018). Notably, while DRD3 were reported not to be expressed in microglial cells, DRD3 deficiency results in attenuated microglial activation upon systemic LPS treatment (Montoya et al., 2019). Hence, the role of DRD3 signaling in the acquisition of inflammatory phenotype by microglial cells was recently further studied by the determination of the M1 and M2 phenotypes acquired by microglia 24 h after LPS treatment in WT and DRD3‐KO mice (Montoya et al., 2019). Interestingly, the percentage of M1 microglia was not affected by genetic deficiency or pharmacological antagonism of DRD3 signaling, but the percentage of M2 phenotype in microglial cells was significantly reduced upon DRD3 antagonism in LPS‐treated WT mice (Montoya et al., 2019). On the bases of these and other results (Elgueta et al., 2017; Montoya et al., 2019), DRD3 has been indicated to be expressed selectively in astrocytes, but not in microglial cells, thereby implicating astrocyte intermediacy in M1‐M2 microglial switch.

#### 1.3.2 DA intersect astrocyte's harmful signaling in PD: DA/αβ‐crystallin /STAT3 crosstalk

**FIGURE 5:**
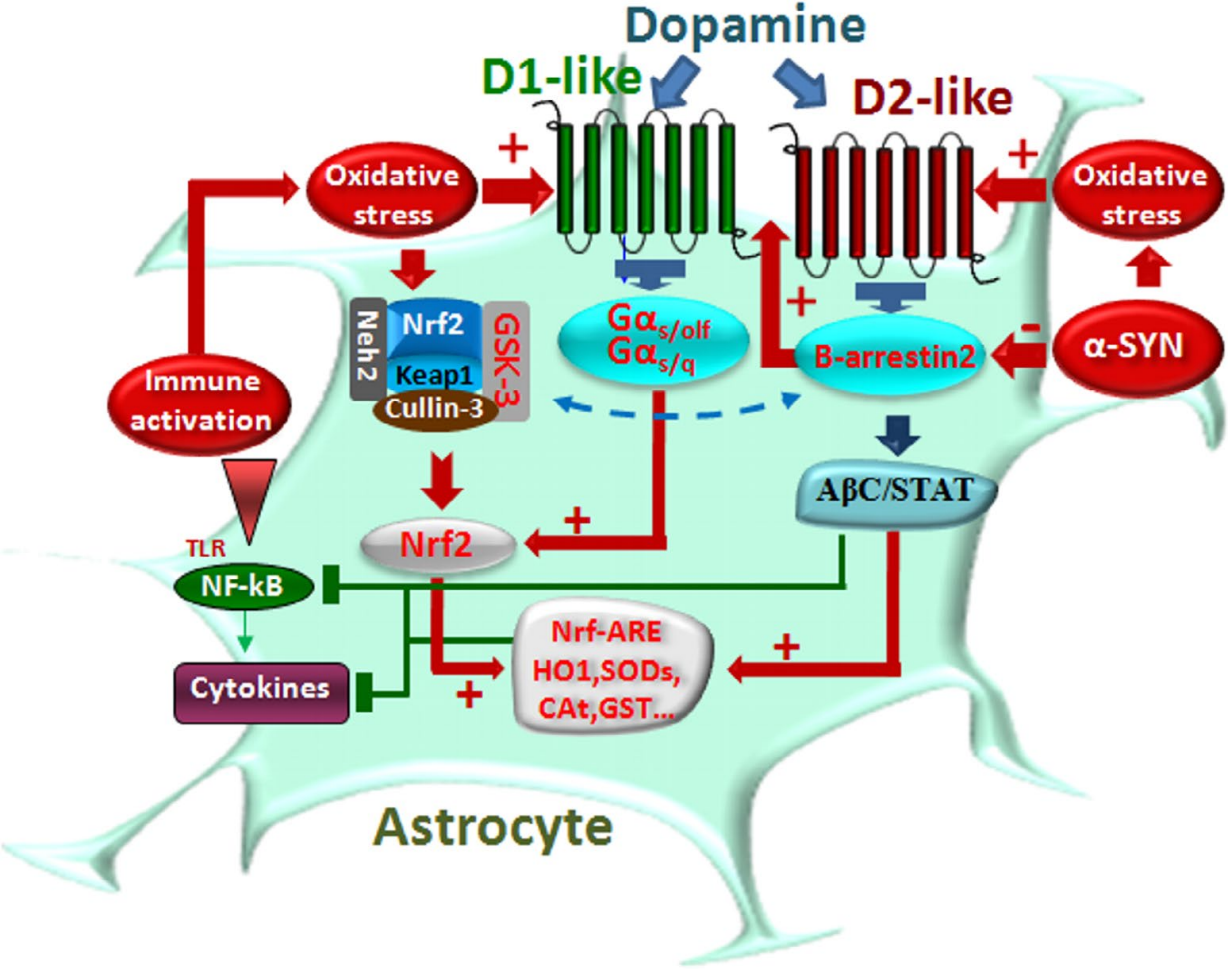
Dopamine signaling pathways intersect oxidative/inflammatory cascades in astrocytes. Schematic representation of DA modulation of astrocyte harmful phenotype during inflammation and oxidative stress. DA crosstalk with Nrf2‐ARE induced targeting of antioxidant response elements (ARE) is highlighted. Upon DA binding to DRD2, neuroinflammation can be mitigated by different mechanisms. αB‐Crystallin (αBC)‐dependent mechanism can be elicited by DRD2 agonists alleviating neuroinflammatory injury via the αβC/STAT3 pathway. DRD2 agonists can also mitigate LPS‐induced proinflammatory cytokine response, via a β‐arrestin‐2‐mediated signaling inhibiting NLRP3 inflammasome activation. On the contrary, α‐Syn reduced the expression of β‐arrestin‐2 in astrocytes, whereas it increased the β‐arrestin‐2 and can restore the anti‐inflammatory effect of DRD2 (detailed in the text). A critical loop is represented by the ability of DRD signaling to upregulate the master regulator of oxidative stress and inflammation, Nrf2 in astrocytes, via ARE stimulation of a panel antioxidant/anti‐inflammatory proteins, such as heme oxygenase (HO1), superoxide dismutases (*SODs*), glutathione S‐transferase (*GST*), and catalase (*CAT*) besides others, regulating the cellular redox state by decreasing oxidative stress and inflammation (detailed in Section 1.4)

Astrocytic DA modulation carried out by DRD2 can suppress neuroinflammation through αB‐crystallin‐dependent mechanism (Figure 5). Hence, DRD2 agonist quinpirole increased resistance of the nigral dopaminergic neurons to MPTP through partial suppression of inflammation (Shao et al., 2013). Conversely, knockout mice lacking DRD2 showed robust inflammatory responses in different brain regions. Additionally, DRD2 knockout increased the vulnerability of mDAns to MPTP‐induced neurotoxicity. Interestingly enough, DRD2‐deficient astrocytes became hyper‐responsive to immune stimuli in the face of a significant decrease in the level of αB‐crystallin (Shao et al., 2013). Further evidence comes from experiments carried out after ablation of DRD2 in astrocytes resulting in a robust activation of astrocytes in SNpc (Shao et al., 2013). Using gain‐of function or loss‐of‐function settings and pharmacological treatments with the selective DRD2 agonist, quinpirole, increased resistance of the SNpc DA neurons to MPTP, through a partial suppression of inflammation. Overall, these studies indicated that astrocytic DRD2 activation physiologically downregulates neuroinflammation in the studied model, via αB‐crystallin‐dependent mechanism, suggesting a potential novel approach aimed at targeting the astrocyte‐mediated innate immune response (Shao et al., 2013). Likewise, in the study of Qiu et al. (2016), sinomenine was shown to activate astrocytic DRD2 receptors, thereby alleviating neuroinflammatory injury via the αβ‐crystallin /STAT3 pathway after ischemic stroke in mice.

A novel interaction between DA and α‐Syn was recently studied by Du et al. (2018). Here, the authors showed that the selective DRD2 agonist quinpirole can suppress inflammation in the midbrain of wild‐type mice, but not in α‐Syn‐overexpressing mice. DRD2 agonists were also capable to significantly mitigate LPS‐induced inflammatory response in astrocytes (Du et al., 2018).

Interestingly, such DRD2‐mediated anti‐inflammatory effect was dependent on β‐arrestin‐2‐mediated signaling, but not on classical G protein pathway. Additionally, α‐Syn reduced the expression of β‐arrestin‐2 in astrocytes, whereas it increased the β‐arrestin‐2 expression and restored the anti‐inflammatory effect of DRD2 in α‐Syn‐induced inflammation. Such α‐Syn‐mediated disruption of DRD2 anti‐inflammatory effect was carried out by inhibiting the association of β‐arrestin‐2 with transforming growth factor‐beta‐activated kinase 1 (TAK1)‐binding protein 1 (TAB1) and promoting TAK1‐TAB1 interaction in astrocytes, underscoring the ability α‐Syn disrupts the function of β‐arrestin‐2 and inflammatory pathways in the pathogenesis of PD (Du et al., 2018). DRD2 agonists were also found to suppress the upregulation of caspase‐1 and IL‐1β expression in primary cultured mouse astrocytes in response to LPS plus ATP‐induced NLRP3 inflammasome activation (Zhu et al., 2018). Furthermore, using the MPTP mouse model of PD, the authors found that DRD2 agonists inhibited NLRP3 inflammasome activation, evidenced by decreased caspase‐1 expression and reduced IL‐1β release in the midbrain of wild‐type mice. Such anti‐inflammasome effect of DRD2 was abolished in β‐arrestin‐2 knockout and β‐arrestin‐2 small interfering RNA‐injected mice, suggesting a critical role of β‐arrestin‐2 in DRD2‐regulated NLRP3 inflammasome activation (Zhu et al., 2018).

On the contrary, the studies of Elgueta et al. (2017) showed a selective DRD3 transcription in astrocytes but not in microglia. Interestingly, D3R selective antagonist PG01037 reduces the acquisition and activation of M1 phenotype microglia, contributing to an anti‐inflammatory effect and displaying a significant therapeutic effect in PD mouse model (Elgueta et al., 2017).

Of note, the DRD3 immunoreactivity in astrocytes is associated with a clustered pattern, resembling the expression pattern observed for those proteins contained in lipid rafts (see Montoya et al., 2019).

Altogether, DA powerfully modulates glial inflammatory responses via both D1 and D2 receptor subtypes, via its intersection within the major proinflammatory circuits. Moreover, DA‐mediated counter‐regulation of immune response may change according to the activation stage and/or the severity of the proinflammatory glial phenotype, thus suggesting that DA‐mediated immunomodulation not only varies according to the DA receptor subtype and operated transduction pathway but also depends on the severity of inflammation and the counter‐modulatory effects elicited by DA crosstalk with key antioxidant/anti‐inflammatory/cytoprotective *Nrf2*/*Wnt* pathways, as discussed below inthe next sections).

### 1.4 DA signaling intersects the Nrf2/Wnt/β‐catenin/GSK‐3β protective axis

#### 1.4.1 DA‐Nrf2 crosstalk

Nrf2 activation‐induced targeting of antioxidant response elements (AREs) in the promoter region of several hundred genes results in the promotion of a wide panel of cytoprotective, anti‐inflammatory and phase 2 proteins, such as heme oxygenase (HO1), NAD(P)H quinone oxidoreductase (*NQO1*), superoxide dismutases (*SOD1*, SOD2), glutathione S‐transferase (*GST*), glutathione peroxidase (*GPx*), glutathione reductase (*GR*), and catalase (*CAT*), which together are capable of regulating the cellular redox state by decreasing ROS. Specifically, Nrf2 has a multifaceted role in mitochondrial function and inflammatory networks (Blackwell et al., 2015; Dinkova‐Kostova & Abramov, 2015; Holmström et al., 2016; Ryoo & Kwak, 2018). Of major importance, Nrf2 induction is primarily observed in non‐neuronal cells. In astrocytes, this inducible mechanism coordinates expression of several cellular defense pathways including the following: detoxification of reactive oxygen/nitrogen species and xenobiotics, GSH synthesis, and generation of NADPH (see Vargas & Johnson, 2009). Notably, Nrf2 is an important player in the pathogenesis of cancer and common inflammatory, age‐dependent, and most neurodegenerative diseases, and its multifunctional role has been emphasized in several earlier and more recent studies and reviews (Abdalkader et al., 2018; Cano et al., 2021; Cuadrado et al., 2019; Dinkova‐Kostova & Abramov, 2015; Johnson et al., 2008; Lastres‐Becker, 2021; Lastres‐Becker et al., 2016; Marchetti, 2020; Strong et al., 2016; Vargas & Johnson, 2009). Activation of Nrf2 in astrocytes protects neurons from a wide array of insults in different *in vitro* and *in vivo* paradigms, including MPTP‐induced mDAn neurotoxicity, whereas Nrf2 deficiency contributes to neuronal death, supporting the role of astrocytes in determining the vulnerability of neurons to noxious stimuli, in particular mDAns (Calkins et al., 2010; Chen et al., 2009; Copple et al., 2010; Gan et al., 2012; Vargas & Johnson, 2009) (Figure 5 and Table 2). Of note, loss of Nrf2 in the presence of α‐syn expression cooperates to aggravate protein aggregation, neuronal death, and inflammation in early‐stage PD (Lastres‐Becker et al., 2012), further highlighting the critical role of gene–environment harmful interactions in PD.

**TABLE 2:** Dopamine signaling at the NRf2/Wnt/β‐catenin/GSK‐3β interface in PD

	REFERENCES
**DA‐Nrf2 Crosstalk**	
DA activates Nrf2‐regulated neuroprotective pathways in astrocytes and meningeal cells. Nrf2‐deficiency and α‐Synuclein expression synergize to aggravate neuronal death and inflammation in PD.	Shih et al. (2007) Lastres‐Becker et al. (2012)
The Nrf2‐ARE axis in astrocytes is neuroprotective.	Vargas et al. (2009)
Aging‐induced DRD2 downmodulation and Nrf2‐ARE pathway disruption in the subventricular zone (SVZ) drives neurogenic impairment in parkinsonian mice via Nrf2/PI3K‐Wnt/β‐catenin dysregulation.	L’Episcopo et al. (2013); Marchetti & Pluchino (2013)
Physiological concentrations of H_2_O_2_ support DA neuronal survival via activation of Nrf2 signaling in glial cells.	Wang et al. (2021)
Icariin attenuates neuroinflammation and exerts DA neuroprotection via an Nrf2‐dependent mechanism. NRF2 activator dimethyl fumarate as an effective therapeutic agent against PD.	Zhang et al. (2019) Lastres‐Becker et al (2016)
Naringenin targets astroglial Nrf2 to support dopaminergic neurons.	Wang et al. (2019)
Emerging roles of glia‐stem/neuroprogenitor crosstalk for dopaminergic neurorestoration in aged parkinsonian brain via Nrf2/Wnt/β‐catenin crosstalk.	Marchetti et al. (2020)
Ellagic acid protects dopamine neurons from rotenone‐induced neurotoxicity via activation of Nrf2 signaling.	Wei et al. (2020)
Activation of p62‐Keap1‐Nrf2 pathway protects 6‐hydroxydopamine‐induced ferroptosis in dopaminergic cells.	Sun et al. (2020)
Nrf2/Wnt cross‐talk in glia‐neuron interactions promotes resilience of dopaminergic neurons in PD.	Marchetti B (2020)
DRD2 interact with β‐catenin through the second and third intracellular loops and inhibit the entry of β‐catenin into the nucleus, leading to an inhibition of the LEF‐1‐dependent transcription. DRD2 regulates Akt and GSK‐3 via Dvl‐3.	Min et al. (2011) Sutton et al. (2012)
Reactive astrocytes via Wnt/β‐catenin signaling and crosstalk with mDAns and microglia link nigrostriatal injury to repair in the MPTP model of PD.	L’Episcopo et al. (2011a; b) Marchetti et al. (2013)
DRD1 activation improves adult hippocampal neurogenesis and exerts anxiolytic and antidepressant‐like effect via activation of Wnt/beta‐catenin pathways in rat model of Parkinson’s disease.	Mishra et al. (2019a)
Neural stem cell grafts in the aged parkinsonian brain reverse aging and MPTP‐induced mDAn toxicity by promoting astroglia‐driven neurorestoration via Wnt/β‐Catenin Signaling.	L’Episcopo et al. (2018)
DRDs activation mitigates mitochondrial dysfunction and oxidative stress to enhance dopaminergic neurogenesis in 6‐OHDA lesioned rats: A role of Wnt signaling.	Mishra et al. (2019a)
DRD1 agonists induce dynamin related protein‐1 inhibition to improve mitochondrial biogenesis and dopaminergic neurogenesis in rat model of PD.	Mishra et al. (2020)
Axin‐2 knockdown promote mitochondrial biogenesis and dopaminergic neurogenesis by regulating Wnt/β‐catenin signaling in rat model of Parkinson's disease.	Singh et al. (2018b)
In aged MPTP‐injured mice intra‐nigral grafts of young astrocytes boost antioxidant self‐defenses and rejuvenates the aged microenvironment via activation of Nrf2‐driven Wnt/β‐Catenin prosurvival axis with consequent mitigation of mDAn degeneration and inflammation.	Serapide et al. (2020)
DRD2‐dependent cross‐talk modulate Wnt3a expression via an evolutionarily‐conserved TCF/LEF site within the Wnt3 promoter. DRD2 signaling modulated cell proliferation and modifies the pathology in a renal ischemia/reperfusion‐injury disease model, via its effects on Wnt/β‐catenin signaling.	Han et al. (2019)
In ageing mice, Wnt/β‐catenin signaling activation can reverse the impaired neurogenesis of the aged PD brain and promote endogenous neurorepair in parkinsonian mice via an integrated crosstalk with Nrf2/ARE axis and modulation of glial inflammatory reaction.	Marchetti et al. (2020)

Abbreviations: 6‐OHDA, six hdroxy‐dopamine; ARE, antioxidant response elements; DVl, Dishevelled; GSK‐3β, glycogen synthase‐3‐kinase β; Keap1, Kelch Like ECH Associated Protein 1; mDANs, midbrain dopaminergic neurons; MPTP, 1‐methyl‐4‐phenyl‐1,2,3,6‐ tetrahydropyridine; Nrf2, Nuclear factor erythroid 2 ‐like 2; PD, Parkinson’s disease; Wnt, Wingless type.

Also, in aged MPTP mice, the old parenchymal astrocytes in VM loose both DRD2 and Nrf2 transcriptional activity, whereas grafting young astrocytes rejuvenates the microenvironment, resulting in a gain of Nrf2 function, as ARE transcriptional activity and mitochondrial beneficial effects are associated with mDAn neurorescue (Serapide et al., 2020). In particular, *in vivo* and *ex vivo* experiments carried out in astrocyte‐grafted aged MPTP mice underscored the ability of “young” astrocyte's grafts to reprogram the aged parenchymal astrocyte metabolic activity, switching mitochondrial dysfunction, in turn resulting in mitigation of ROS, RNS, and inflammatory mediators, compared with aged MPTP control astrocytes transplanted with a non‐specific cell type (Serapide et al., 2020).

Further, the activation of Nrf2 enables protection against 6‐hydroxydopamine‐(6‐OHDA)‐induced ferroptosis, a form of cell death involving the iron‐dependent accumulation of GSH depletion and lipid peroxide in DA cells (Sun et al., 2020; Wei et al., 2020). By contrast, Nrf2 deficiency was associated with exaggerated mitochondrial dysfunction and blockade of Nrf2’s mitochondrial protective response, as recently reported by Cano et al. (2021) in Nrf2‐deficient retinal pigmented epithelium. The pivotal function of Nrf2 stems from its modulatory role on key aspects of mitochondrial health (see Ammal Kaidery et al., 2019; Cano et al., 2021; Ryoo & Kwak, 2018). Interestingly, in Caenorhabditis elegans, where the Nrf proteins are represented by their ortholog SKN‐1, recent studies implicate Nrf/SKN‐1 in a wide range of homeostatic functions (Blackwell et al., 2015). Reportedly, as underscored by Blackwell et al. (2015), “SKN‐1 plays a central role in diverse genetic and pharmacological interventions that promote C. elegans longevity, suggesting that mechanisms regulated by SKN‐1 may be of conserved importance in aging” (Blackwell et al., 2015). Accordingly, a number of experimental approaches evaluating the potential regulation of the transcription factor Nrf2 to enhance the expression of genes that contrast oxidative stress and promote healthy aging have been provided, particularly with Nrf2 activators described to expand the life span, contrasting oxidative stress and inflammation (Liu et al., 2009; Nelson et al., 2006; Strong et al., 2016; Velmurugan et al., 2009).

Against this background, a direct DA‐Nrf2 crosstalk may represent a further protective mechanism whereby DA activation triggers Nrf2‐regulated pathways (Figure 5). Hence, in astrocytes, excessive extracellular DA itself likely served as an endogenous signal to activate Nrf2‐dependent neuroprotective pathways (Shih et al., 2007). Indeed, the ability of Nrf2 activation in protecting cells from DA toxicity has long been recognized, and in part attributable to enhanced H_2_O_2_ scavenging by the GSH system, and detoxification of reactive quinones by NAD(P)H: NQO1 (Duffy et al., 2018). Particularly, physiological oxidative stressors or subthreshold concentrations of neurotoxins support DA neuron survival and neural stem progenitor cell (NSC) differentiation via activation of Nrf2/Wnt signaling in glial cells (Marchetti et al., 2020; Wang et al., 2020). Notably, DA activation of DRD1‐like receptor, DRD5, was recognized as a necessary trigger for the normal expression of Nrf2 and inhibition of harmful oxidative cascades, as DRD5 deficiency causes an increase in NADPH oxidase activity and prevents the translocation of Nrf2 nuclear (Jiang et al., 2018). Then, DA activation of D1‐like receptors in astrocytes might further contribute to an autoregulatory feedback triggered by endogenous DA and DA agonists (Figure 5, Table 2).

Together, DA‐Nrf2 crosstalk appears a feasible counter‐regulatory mechanism triggered by DA to prevent the deleterious effects of exacerbated oxidative stress and inflammation.

#### 1.4.2 DA‐Wnt/β‐catenin crosstalk

Earlier studies on functional interactions between DA and Wnt/β‐catenin signaling focused on DRD2 under long‐term treatment with antipsychotic drugs, which are the blockers of D2‐like receptors (Alimohamad et al., 2005; Freyberg et al., 2010), supporting a functional interaction between Wnt pathway and DRD2/DRD3. The chief role of Wnt signaling for neurogenesis in the adult and aged PD brain has been recently reviewed (Marchetti et al., 2020). During age and basal ganglia injury, the progressive decline in DA targeting glial cells via DRD2 in VM and Str of aged MPTP‐treated PD mice was associated with decreased Wnt/β‐catenin signaling genes and proteins, in turn affecting both glial cell reactivity and mDAn loss (Marchetti, 2018). Moreover, decreased D1 receptor expression, mitochondrial biogenesis, mitochondrial functions, and dopaminergic neuron differentiation were associated with downregulation of Wnt/β‐catenin signaling in the hippocampus of rats lesioned with the PD neurotoxin, 6‐OHDA (Mishra, Singh, Tiwari, Chaturvedi, et al., 2019; Mishra, Singh, Tiwari, Parul, et al., 2019). Conversely, pharmacological stimulation of D1 receptor enhanced mitochondrial biogenesis, mitochondrial functions, and DA neurogenesis that lead to improved motor functions in 6‐OHDA‐injured rats. The specificity of these effects was underscored using a D1 antagonist, whereas shRNA‐mediated knockdown of Axin‐2, a negative regulator of Wnt signaling, significantly abolished D1 antagonist‐induced impairment in mitochondrial biogenesis and DA neurogenesis in 6‐OHDA‐lesioned rats (Mishra, Singh, Tiwari, Chaturvedi, et al., 2019; Mishra, Singh, Tiwari, Parul, et al., 2019).

**FIGURE 6:**
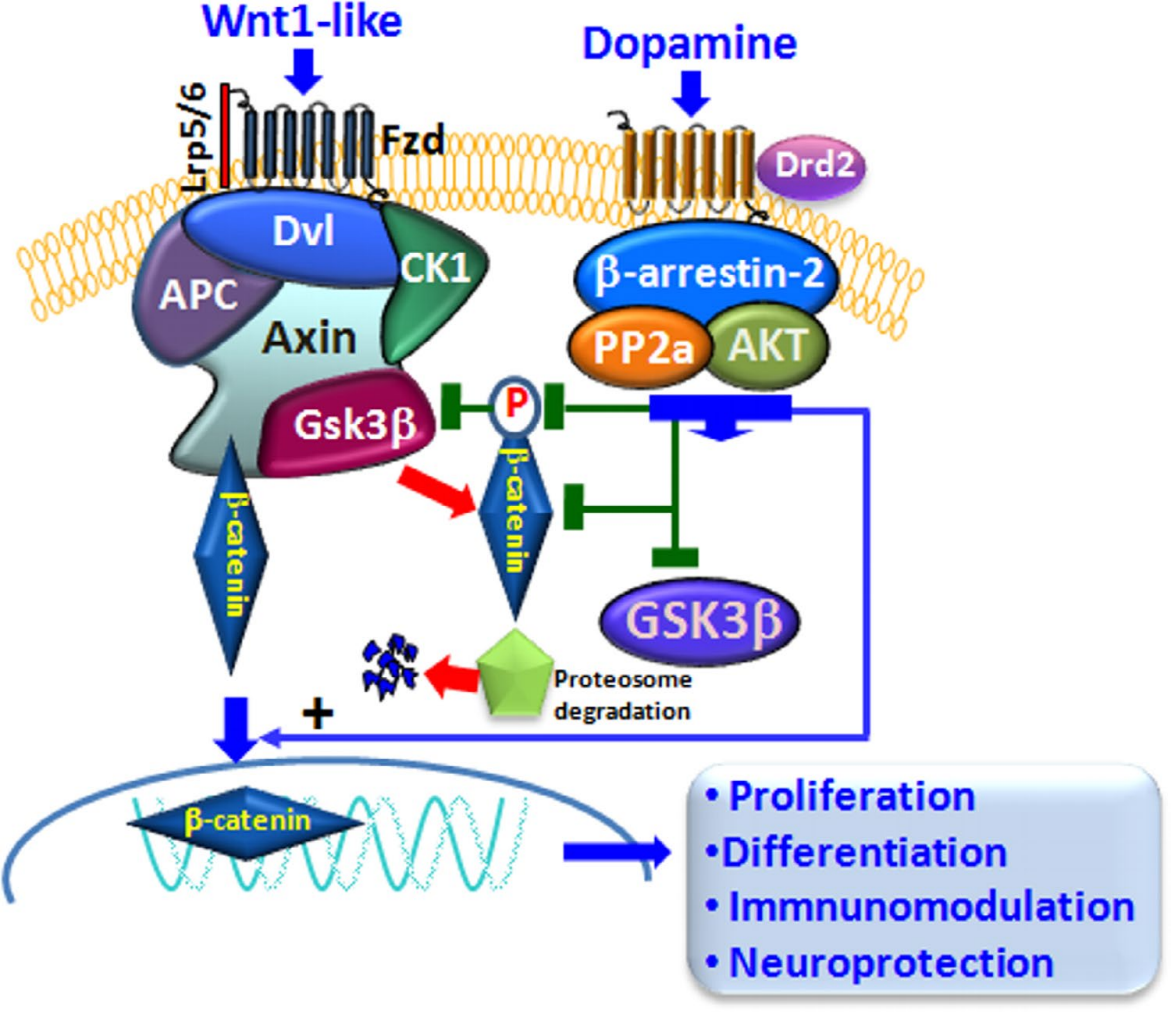
Dopamine signaling pathways crosstalk with Wnt/β‐catenin/GSK‐3β cascade. Simplified representation of the Wnt/β‐catenin signaling pathway and its intersection with DRD2 signaling. Wnt signal activation is tightly controlled by a dynamic signaling complex comprised of core receptors from the Frizzled (Fzds) family of G protein‐coupled receptors (GPCRs), the low‐density lipoprotein (LDL) receptor‐related protein (LRP) 5/6 co‐receptors, and the disheveled (Dvl) and Axin adapters. Binding of Wnt1‐like endogenous/exogenous agonists to Fzd triggers a molecular cascade leading to the cytoplasmic accumulation of β‐catenin, which enters the nucleus, and associates with T‐cell factor/lymphoid enhancer binding factor (TCF/LEF) transcription factors, in turn promoting the transcription of Wnt target genes. β‐Catenin is tightly regulated via phosphorylation by the ‘destruction complex’, consisting of glycogen synthase kinase‐3β (GSK‐3β), casein kinase 1α (CK1α), the scaffold protein Axin, and the tumor suppressor adenomatous polyposis coli (APC). DRD2 downstream intracellular G protein‐independent, arrestin‐dependent pathways can target Wnt/β‐catenin signaling, intersecting GSK‐3β, through the contribution serine/threonine kinase (AKT)‐mediated phosphorylation. Crosstalk between DRD2 and Wnt signaling can relieve β‐catenin from active GSK‐3 phosphorylation, thus permitting β‐catenin translocation in the nucleus activating transcription of Wnt‐dependent genes involved in proliferation, differentiation, neuroprotection and immunomodulation (detailed in the text)

A number of studies investigated the molecular mechanisms of DRD2‐Wnt/β‐catenin crosstalk (Han et al., 2019; Min et al., 2011). In the study of Min et al. (2011), among the five DA subtypes, DRD2 interacted with β‐catenin through the second and third intracellular loops and inhibited the entry of β‐catenin into the nucleus, leading to an inhibition of the LEF‐1‐dependent transcription (Min et al., 2011). In this work, the authors suggested that the functional regulation of Wnt signaling by DRD2 could occur through direct interaction with β‐catenin independently of the upstream signaling components (Min et al., 2011). Notably, of the two DRD2 downstream intracellular pathways, the β‐*arrestin*‐*dependent* pathway appears to be the one targeting Wnt/β‐catenin signaling (Bryja et al., 2007), with GSK‐3β, being the critical intersector, and the contribution of serine/threonine kinase (AKT) counter‐regulation (Figure 6).

In fact, in addition to AKT’s roles in β‐arrestin‐2‐dependent DRD2 signaling, AKT regulates GSK‐3β through phosphorylation. In its non‐phosphorylated state, GSK‐3β is constitutively active, whereas AKT‐induced phosphorylation inactivates GSK‐3β (Beaulieu et al., 2007) (Figure 6). Regarding the so‐called “canonical Wnt/β‐catenin” signaling, GSK‐3β is part of a destruction complex, whereby GSK‐3β‐induced phosphorylation of β‐catenin results in its proteasomal degradation, blockade of β‐catenin nuclear translocation associated with inhibition of Wnt‐dependent transcription of a panel of downstream target genes. Then, DA activation of DRD2‐β‐arrestin‐2‐dependent pathway may also modulate Wnt signaling via AKT‐mediated phosphorylation of GSK‐3β, thereby modulating β‐catenin nuclear translocation (Figure 6).

Significantly, in the study of Han et al. (2019), DRD2‐dependent crosstalk was shown to modulate Wnt3a expression via an evolutionarily conserved TCF/LEF site within the Wnt3 promoter. Moreover, DRD2 signaling also modulated cell proliferation and modifies the pathology in a renal ischemia/reperfusion injury disease model, via its effects on Wnt/β‐catenin signaling, thus suggesting DRD2 as a transcriptional modulator of Wnt/β‐catenin signal transduction, with broad implications for health and development of new therapeutics (Han et al., 2019).

Importantly, DRD2‐mediated Wnt‐β‐catenin signaling also crosstalks with major immune signaling actors. Hence, if not phosphorylated by GSK‐3β, β‐catenin forms a complex with both the units of NF‐κB, altering its DNA binding activity, and consequently inhibits the inflammatory cascade (Marchetti & Pluchino, 2013). However, when GSK‐3β is activated, it phosphorylates β‐catenin protein for proteasomal degradation that directly promotes the inflammatory events (Deng et al., 2002; Marchetti & Pluchino, 2013). Activated GSK‐3 also modulates CREB‐DNA activity, phosphorylating NF‐κB, and degrades β‐catenin, thus promoting systemic inflammation.

The ability of active GSK‐3β to phosphorylate Nrf2 (Cuadrado et al., 2018; Hayes et al., 2015) then represents a further vulnerability factor, as its overexpression exacerbates inflammation, thus impairing neuron–glial and glial–NSC interactions leading to enhanced neuronal vulnerability and/or cell death, associated with reduced neurorepair (Marchetti, 2020). By contrast, DA‐activated DRD2‐β‐arrestin‐2‐dependent signaling via AKT can boost the antioxidant, anti‐inflammatory, prosurvival, and neurogenic downstream gene cascade.

**FIGURE 7:**
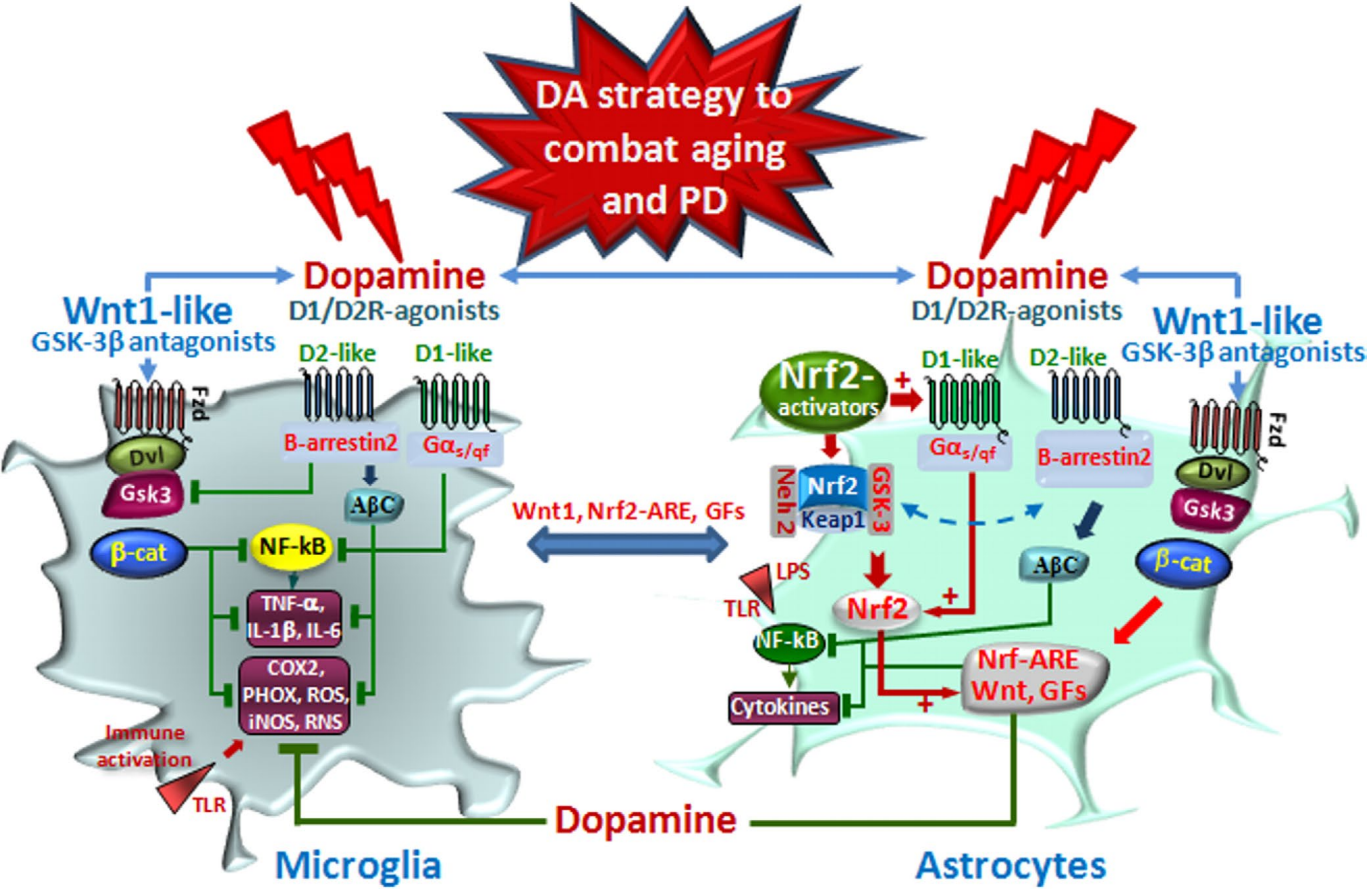
Dopamine drives astrocyte–microglial crosstalk via DRDs/Nrf2/Wnt/GSK‐3 signaling to combat oxidative stress and inflammation. Schematic representation of DA signaling pathways intersecting major oxidative/inflammatory networks in astrocyte–microglial dialogue in PD. Aging, inflammation, and toxic (including bacterial, viral, neurotoxic…) exposures work in synergy with genetic mutations impair nigrostriatal DA neurons. DA and DA agonist can revert such harmful dialogue via a glial switch toward a beneficial antioxidant/anti‐inflammatory and neuroprotective phenotype. DA and DA agonists acting via DRD1 and DRD2 in astrocytes can upregulate *Nrf2*/*HO1* and *Wnt1*/*β*‐*catenin* during oxidative stress and inflammation representing a self‐defense system for mDAn survival. Increased DRD2‐β‐arrestin‐2/AKT cascade may then block GSK‐3β‐induced phosphorylation and proteasomal degradation of the neuronal pool of β‐catenin. Stabilized β‐catenin can translocate into the nucleus and associate with a family of transcription factors and regulate the expression of Wnt target genes involved in DA neuron survival/plasticity, neuroprotection and repair. Oxidative stress engendered by DA itself may also function as a critical negative feedback mechanism via DRD5 induction Nrf2‐ARE cascade and/or via DRD2/β‐arrestin‐2‐induced GSK‐3 inhibition, leading to Nrf2 nuclear translocation. DA‐induced beneficial astrocyte phenotype also intersects microglial inflammatory phenotype via both direct DRD1 and DRD2 transduction pathways inhibiting NLRP3/ NF‐ĸB cascade, and/or via astrocyte beneficial feedback onto microglial cells, via astrocytic Wnt1‐like ligands through Fzd receptors, GSK‐3β antagonist, or HO1‐induced anti‐inflammatory effects

As a whole, DA‐mediated signaling at the astrocyte–microglial interface via DRDs appears as a pivotal counter‐regulatory system contributing to limit both *Nrf2* and *β*‐*catenin* phosphorylation and subsequent degradation, thereby reinforcing the Nrf2‐ARE/Wnt/β‐catenin neuroprotective and immunomodulatory axis to combat aging and PD (Figure 7), and can be envisaged for the treatment of other CNS diseases.

### 1.5 “Reframing” DA agonists as immune modifiers in CNS disorders

Accordingly, increasing evidence suggests the potential to redirect DA drugs to downregulate inflammation at both central and peripheral levels, with the ability of well‐recognized indirect and direct DA agonists (including levodopa, pramipexole, ropinirole, quinpirole, apomorphine, and amantadine among others), used in the symptomatic therapy of PD, to be repurposed by virtue of their neuroprotective/anti‐inflammatory properties in a wide panel of disorders, including ischemic stroke, intracerebral hemorrhage, ICH, traumatic brain injury, TBI, and amyotrophic lateral sclerosis, ALS, besides others. Hence, studies of Yan et al. (2015) showed that DA prevented NLRP3‐dependent neuroinflammation via regulating dopamine DRD1/cAMP signaling pathway and suggested D1R agonists as potential therapeutic target for the inflammation‐related CNS diseases (Yan et al., 2015). The work of Zhang et al. (2015) implicated DRD2 agonists in suppressing neuroinflammation via αB‐crystallin, via inhibition of NF‐κB nuclear translocation in experimental ICH mouse model. The role of DA in poststroke inflammation has been deeply studied by several investigators. Reportedly, levodopa treatment improves functional recovery after experimental stroke (Ruscher et al., 2012) both directly and via DRD‐induced glial cell line‐derived neurotrophic factor (Kuric et al., 2013), with the role of DA–immune cell signaling in poststroke inflammation expanded by Talhada et al. (2018). Also, DRD2 agonist, bromocriptine methylate, can suppress glial inflammation, thus mitigating disease progression in a mouse model of ALS (Tanaka et al., 2011), and quinpirole‐mediated regulation of DRD2 can inhibit glial neuroinflammation both in the cortex and in the Str after TBI (Alam et al., 2021). Notably, the studies of Wang et al. (2018) showed the ability of DRD1 activators to decrease NLRP3‐mediated inflammation in ICH, and in a rat model of spinal cord injury, the DRD1 agonist, A‐68930, inhibits NLRP3 activation‐mediated inflammation and alleviates histopathology (Jiang et al., 2015).

**FIGURE 8:**
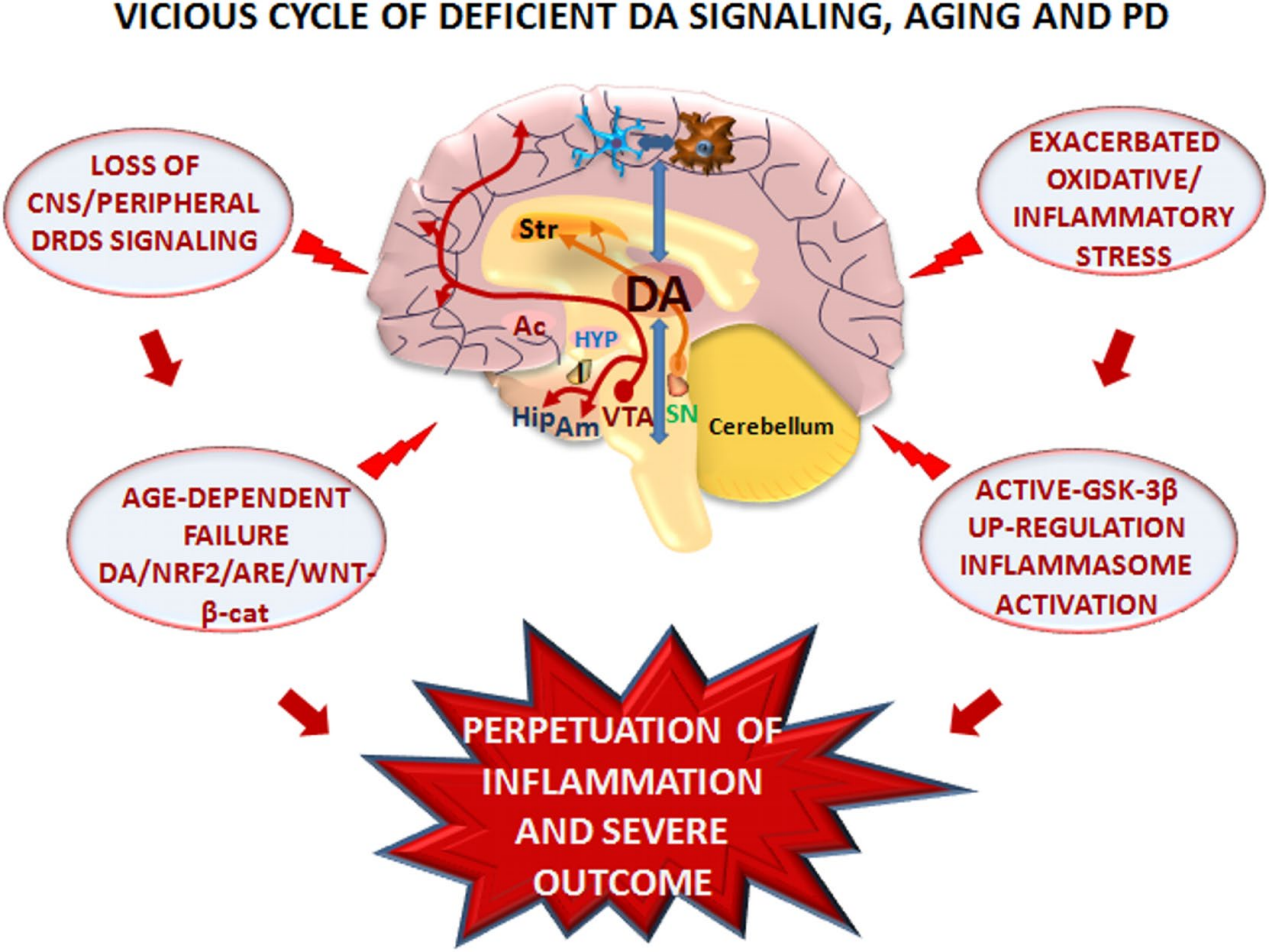
Vicious cycle of dopamine deficiency, aging, inflammation, and CNS disease. Schematic illustration of the interacting harmful cascades arising from DA deficiency at central and peripheral levels engendering a detrimental vicious cycle. The dramatic loss of DA‐mediated signaling at central and peripheral levels associated with the age‐ and PD‐dependent GSK‐3β overactivation in turn creates a favorable milieu driving a feedforward cycle of inflammation/neurodegeneration, as loss of Nrf2/Wnt and upregulation of GSK‐3 phosphorylating and degrading β‐catenin further drive inflammation and excessive oxidative stress associated with inhibition of adult neurogenesis and neurorepair (Marchetti, 2020)

Altogether, DA agonists emerge as potential therapeutics in a wide number of CNS diseases, in as much as the interacting harmful cascades arising from DA deficiency at central and peripheral levels, may engender a detrimental vicious cycle (Figure 8). Hence, the dramatic loss of DA‐mediated signaling at central and peripheral levels was associated with the age‐dependent GSK‐3β overactivation, in turn creating a favorable milieu driving a feedforward cycle of inflammation/neurodegeneration, as loss of Nrf2/Wnt and upregulation of GSK‐3 phosphorylating and degrading β‐catenin further drive inflammation and excessive oxidative stress, which is linked to the inhibition of adult neurogenesis and neurorepair (Marchetti, 2020; Marchetti et al., 2020).

Notably, a panel of “protective/beneficial” strategies targeting lifestyle, such as physical activity and exercising, diet and dietary supplementations/restrictions, impacting on global organic/psychomental health and abating the harmful effects of chronic stress, have resilient effects on Nrf2/ARE‐Wnt/β‐catenin axis (Marchetti, 2020) and may promote a more resistant phenotype (Figure 8).

## 2 CONCLUDING REMARKS AND FUTURE PERSPECTIVES

Oxidative stress and inflammation are recognized aggravating factors for the development of both the sporadic and the genetic forms of PD, where the exacerbated generation ROS and a panel of proinflammatory cytokines targeting mDAn mitochondria contribute to the progressive dysfunction and death of nigrostriatal neurons. Here, we highlighted DA and its signaling pathways, intersecting astrocyte–microglial oxidative/inflammatory networks in PD vulnerability. Notably, the intrinsic propensity of DA neurons to oxidative stress and glial inflammatory triggers, coupled to the aging process and a genetic predisposition, dictates the vulnerability to PD. Importantly, DA emerges as a novel critical modulator of astrocytes and microglial reactivity, as well as systemic inflammation thanks to the expression of specific classes of DA receptors, in both central and peripheral immune cells and intermingled crosstalk with Nrf2/Wnt/β‐catenin cascades.

Hence, in reviewing the pivotal role of DA in controlling the harmful consequences of oxidative stress and inflammation, we introduce a novel perspective underscoring DA’s ability to serve as an endogenous signal to activate Nrf2‐dependent antioxidant, metabolic cytoprotective pathways. In turn, glial activation engenders a DA autoregulatory feedback loop via DRs upregulation to provide a counter‐regulatory mechanism. Within this frame, DA activation of DRD2/β‐arrestin‐2‐dependent pathway may also modulate Wnt signaling via AKT‐mediated inactivation of GSK‐3β, thereby favoring β‐catenin nuclear translocation and the transcription of a panel of Wnt‐dependent prosurvival and anti‐inflammatory genes.

Aside PD, increasing evidence also suggests the potential to redirect DA drugs to downregulate inflammation at both central and peripheral levels, with the ability of well‐recognized indirect and direct DA agonists used in the symptomatic therapy of PD to be reframed by virtue of their neuroprotective/anti‐inflammatory, and herein described as potential positive modulators of the resilient Nrf2/Wnt axis, in a wide panel of disorders.

As a whole, novel perspectives can be envisaged for the therapeutic management of both central and peripheral disorders, where inflammation and oxidative stress represent the core of a self‐perpetrating age‐dependent disease, with relevance for developing novel therapeutic options for NDs. Against this background, different challenges and questions still remain open, and much has to be further disclosed regarding the aging process and what can be translatable to age‐related functional decline in humans in order to be relevant for aging research and drug discovery as well as for rational therapeutics as recently underscored (Bakula et al., 2019; Evans et al., 2021; Gorgoulis et al., 2019; Mkrtchyan et al., 2020; Zhu et al., 2021).

Significantly, the tremendous growth of the elderly population, coupled to the emerging role of viral infections that will further increase worldwide, represents a unique challenge for the development of integrated therapies, drug repurposing, and redirection of “old” drugs, using high‐throughput analysis to identify most effective drug candidates, establishing novel multiorgan‐on‐a‐chip systems for drug discovery platforms, besides others, but especially for the discovery of robust disease biomarkers, to prevent and/or combat “harmful” aging and PD.

## CONFLICT OF INTEREST

We declare no conflict of interest.

## AUTHOR CONTRIBUTIONS

BM conceptualized the study, wrote the manuscript, and gave final approval of the submitted material. MFS, CG, and CT contributed to original artwork for all Figures, provision of study material and bibliography, manuscript editing, and final manuscript approval.

## Data Availability

This work has been supported by grants of the Italian Agencies, Italian Ministery of Health (Ricerca Corrente 2018‐2022) and Italian Ministery of University Research (“Bando‐Chance”, PRIN‐2015). The support of the OASI institute‐IRCCS‐ of Troina (EN), particularly the Laboratory of Neuropharmacology, and BIOMETEC (PIACERI, University of Catania) are acknowledged.
